# Visualized podocyte-targeting and focused ultrasound responsive glucocorticoid nano-delivery system against immune-associated nephropathy without glucocorticoid side effect

**DOI:** 10.7150/thno.53083

**Published:** 2021-01-01

**Authors:** Kui Fan, Li Zeng, Jing Guo, Shuqin Xie, Yuan Yu, Jianwei Chen, Jin Cao, Qinyanqiu Xiang, Siliang Zhang, Yuanli Luo, Qingyue Deng, Qin Zhou, Yan Zhao, Lan Hao, Zhigang Wang, Ling Zhong

**Affiliations:** 1 Department of Nephrology, The Second Affiliated Hospital of Chongqing Medical University, Chongqing 400010, China.; 2 Institute of Ultrasound Imaging, The Second Affiliated Hospital of Chongqing Medical University, Chongqing 400010, China.; 3 Department of Nephrology, Santai County People's Hospital (Affiliated Hospital of North Sichuan Medical College in Santai County), Mianyang 621100, China.; 4 State Key Laboratory of Ultrasound in Medicine and Engineering, Chongqing Medical University, Chongqing 400016, China.; 5 Radiation Oncology Center, Chongqing University Cancer Hospital, Chongqing University, Chongqing 400030, China.; 6 Department of Respiratory Medicine, The Second Affiliated Hospital of Chongqing Medical University, Chongqing 400010, China.

**Keywords:** immune-associated nephropathy, podocyte, dexamethasone, nano-delivery system, side effect

## Abstract

Glucocorticoids are widely used in the treatment of nephritis, however, its dose-dependent side effects, such as the increased risk of infection and metabolic disturbances, hamper its clinical use. This study reports a visualized podocyte-targeting and focused ultrasound responsive glucocorticoid nano-delivery system (named as Dex/PFP@LIPs-BMS-α), which specific delivers dexamethasone (Dex) to podocyte targets and reduces systemic side effects.

**Methods:** The glucocorticoid nano-delivery system was synthesized by a lipid thin film and a simple facile acoustic-emulsification method. This glucocorticoid nano-delivery system used BMS-470539 (BMS-α), a synthetic compound, as a “navigator” to specifically identify and target the melanocortin-1 receptor (MC-1R) on podocytes. The loaded perfluoropentane (PFP) realizes the directed "explosion effect" through ultrasound-targeted microbubble destruction (UTMD) technology under the coordination of low intensity focused ultrasound (LIFU) to completely release Dex.

**Results:** Both *in vitro* and *in vivo* experiments have demonstrated that Dex/PFP@LIPs-BMs-α accurately gathered to podocyte targets and improved podocyte morphology. Moreover, *in vivo*, proteinuria and serum creatinine levels were significantly reduced in the group treated with Dex/PFP@LIPs-BMS-α, and no severe side effects were detected. Furthermore, Dex/PFP@LIPs-BMS-α, with capabilities of ultrasound, photoacoustic and fluorescence imaging, provided individualized visual guidance and the monitoring of treatment.

**Conclusion:** This study provides a promising strategy of Dex/PFP@LIPs-BMS-α as effective and safe against immune-associated nephropathy.

## Introduction

Immune-associated nephropathy is the leading cause of kidney disease. Podocytopathies is the characterize of immune-associated nephropathy, such as Idiopathic Membranous Nephropathy (IMN) 1, Lupus Nephritis (LN) 2, Immunoglobulin A Nephropathy (IgAN) 3, and Minimal Change Disease (MCD) 4. Podocytes are highly specialized epithelial cells in the kidneys involved in maintaining glomerular filtration. Impairment of podocyte function results in proteinuria, progression of nephrotic syndrome, resulting in development of end-stage renal disease (ESRD) 5-9. Many factors are involved in the damage of podocytes that result in morphologic and functional changes of podocytes. These include actin cytoskeletal rearrangement, loss of slit diaphragm structure and reduced glomerular basement membrane interaction with podocytes. Under stress adaptive morphological changes of podocytes may mitigate decreases in renal function, the therapeutic value of stabilizing actin cytoskeleton has been demonstrated 10,11.

Glucocorticoids are the first-line therapy for patients with immune-associated nephropathy. In studies of cultured human and rat podocytes, dexamethasone (Dex) was demonstrated to stabilize actin cytoskeleton and directly protect podocytes by inhibiting the breakdown of actin by regulated cyclic guanosine monophosphate in the cGMP-PKG pathway 12-14. Dex acts through expression of glucocorticoid receptors on podocytes 15. This action suppresses complement activation of in situ circulating immune-complexes in the kidney, and prevents further podocyte injury and consequent proteinuria induced by mediators that would have been produced by podocytes, harmfully stimulated by complement membrane attack complex. This mechanism strongly supports the role of glucocorticoids in the management of clinical nephritis. However, the virtually inevitable side effects of systemic glucocorticoids, including an increased risk of infection and metabolic disturbances such as hyperglycemia, even severe enough to be fatal, greatly limit its clinical utilization 15. Although there have been reports of medical engineering technology to develop prodrug and macromolecular vehicles-based, drug-targeted delivery systems to target kidneys in order to reduce side effects. One example was a polyethylene glycol-based macromolecular prodrug of Dex, this was based on a passive inflammation-targeting mechanism and pH-sensitive drug release, but did not have visual imaging diagnosis or treatment functions 16. Yet another, Dex was delivered based on extracellular vesicles, but this system did not have either controlled drug release or visual imaging functions 17. Based on omics research to improve diagnostic performance and treatment response 18,19. Although study has shown that melanocortin-1 receptor (MC-1R) has polymorphisms differentially alter multiple signaling pathways through omics methods, the affinity between BMS-α and MC-1R is very high 20. The objective of this study was to design a visualized podocyte-targeting technology, based on MC-1R, and focused ultrasound responsive glucocorticoid nano-delivery system of Dex/PFP@LIPs-BMS-α with targeted drug distribution, to demonstrate efficacy of treatment, and to minimize the systemic adverse glucocorticoid side effects.

To specifically target delivery of Dex to podocyte targets, MC-1R, which is expressed on podocytes, and is highly expressed in the kidney 21. BMS-470539 (BMS-α) is a highly selective agonist of MC-1R without activating any other melanocortin receptors 22,23. Although there were doubts expressed regarding use of foreign targeted ligands leading to harmful immune responses 17, BMS-α combined with MC-1R regulates transcription factors through the cAMP-PKA pathway, which can stabilize and repair the podocyte actin cytoskeleton 23-25. Therefore, the use of BMS-α as the target ligand of the drug delivery system was intended to provide precise targeting, avoid unexpected immune responses, and result in the improvement of both the morphology and function of podocytes.

Liposomes are widely used as vehicles of drug delivery systems due to their favorable biocompatibility, high drug loading rate and high biosafety 26-28. Our previous work, delivering antitumor drugs, via liposomes, to metastatic clear cell renal cell carcinoma, confirmed these properties of the liposome-based drug delivery system 29. Furthermore, liposome-based drug delivery systems have been used for treating type 2 diabetes and methotrexate-induced kidney injury 30,31. Simple nanoliposomes-based microspheres can effectively penetrate the renal cell barrier, have satisfactory *in vitro* imaging capability but they have uncontrollable drug release 32,33. We loaded Perfluoropentane (PFP) inside the nanoliposomes-based microspheres, then acoustic droplet vaporization (ADV) effect and ultrasound targeted microbubble destruction (UTMD) technology were induced by low intensity focused ultrasound (LIFU) to achieve controlled drug release and *in vitro* imaging 34-39.

**Scheme 1:**
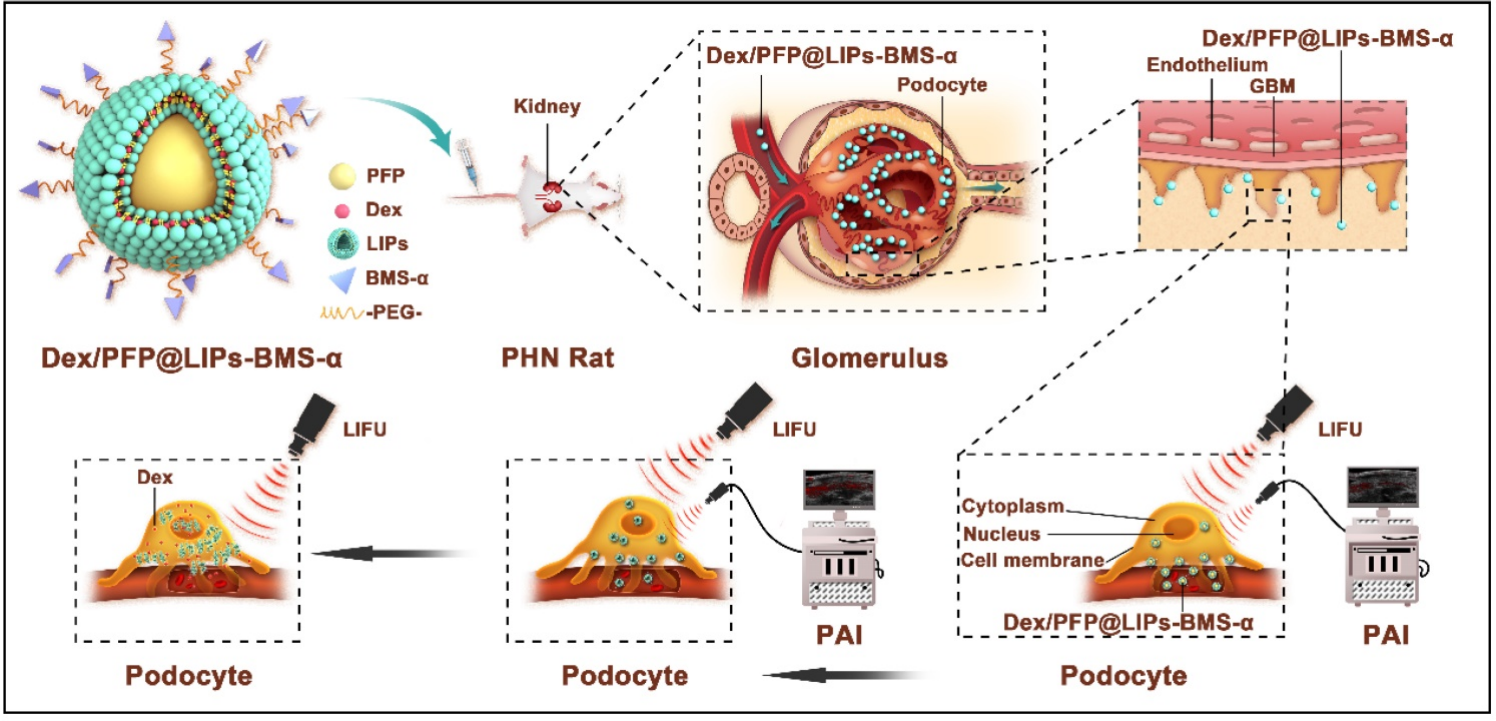
**Schematic illustration of the structure of Dex/PFP@LIPs-BMS-α, and the theranostic functions of Dex/PFP@LIPs-BMS-α.** Liposomes (LIPs) were the main carrier of Dex/PFP@LIPs-BMS-α, which was internally loaded with PFP and Dex, and externally connected with BMS-α by polyethylene glycol (PEG). Glucocorticoid nano-delivery systems were injected into PHN rats through tail vein, which could specifically identify and target binding of MC-1R. Then, the target organ was irradiated by LIFU to fully delivery and release Dex to against PHN.

A new therapeutic strategy is proposed for immune-associate nephritis by targeting podocytes and evaluation of the treatment effectiveness. As shown in **Scheme 1**, the visualized podocyte-targeting and focused ultrasound responsive glucocorticoid nano-delivery system was loaded with Dex and PFP inside and connected BMS-α by polyethylene glycol (PEG). First, we used PEG to facilitate delivery of the bio-complex, protecting against recognition and destruction by the immune system, that allowed release and activation of antigen-specific T-cell 40,41. Moreover, we confirmed that Dex/PFP@LIPs-BMS-α specifically close proximity to the podocytes via the navigation by BMS-α and subsequent therapy by LIFU *in vivo* and *in vitro*. Third, the highly specific targeting of near podocytes and the precise targeted release of Dex effectively avoided systemic, dose-dependent immunosuppression and additional side effects caused by Dex. Furthermore, Dex/PFP@LIPs-BMS-α was triggered by LIFU or it was fluorescently labeled, giving it outstanding ultrasonic, photoacoustic or fluorescence imaging capabilities. These results demonstrated that Dex/PFP@LIPs-BMS-α was a promising multifunctional theranostic agent in immune-associated nephropathy therapy.

## Methods

### Materials and reagents

1,2-dipalmitoyl-sn-glycero-3-phosphatidylcholine (DPPC), 1,2-dipalmitoyl-sn-glycero-3-phospho-(1-rac-glycerol) (DPPG), and cholesterol (Chol) were purchased from Avanti Polar Lipids, Inc. (Alabaster, AL, USA). DSPE-mPEG_2000_-COOH (DSPE-PEG-COOH) was purchased from Ruixi Biotechnology (Xi'an, China). Perfluoropentane (PFP) was purchased from Apollo Scientific Ltd (Chesshire, UK). BMS-470539 dihydrochloride (BMS-α) was obtained from APExBIO Technology LLC, Inc. (Houston, TX, USA). 1,1'-dioctadecyl-3,3,3',3'tetramethylindocarbocyanine perchlorate (DiI), 2-(4-amidinophenyl)-6-indolecarbamidinedihydrochloride (DAPI) and 1,1'-dioctadecyl-3,3,3',3'-tetramethylindotricarbocyanine iodide (DiR) were obtained from Sigma-Aldrich (Saint Louis, MO, USA). Dexamethasone (Dex) and dexamethasone sodium phosphate (DSP) were obtained from Beijing Solarbio Science & Technology Co., Ltd. (Beijing, China). Puromycin aminonucleoside (PA, Sigma-Aldrich, Stockholm, Sweden). Cell counting kit-8 (CCK-8, ab228554), anti-MC1R antibody (MC-1R, ab236734/ab180776), goat anti-rabbit IgG H&L (Alexa Fluor® 488, ab150077), DCFDA cellular ROS detection assay kit (DCFDA, ab 113851) and rabbit anti-CD3 antibody (ab5690) were obtained from abcam Inc. (Cambridge, MA, USA). TRITC-phalloidin was obtained from Yeasem Biotechnology Co., Ltd. (Shanghai, China). Sheep anti-rat Fx1A serum (anti-Fx1A) was obtained from Probetex Inc. (San Antonio, TX, USA). All other reagents were of analytical grade and shall be used as required.

### Preparation of Dex/PFP@LIPs-BMS-α

Conjugation of BMS-α to DSPE-PEG-COOH by the carbodiimide method. Briefly, 80 mg DSPE-PEG-COOH were dissolved in a mixture of 20 mL N'N-dimethyl formamide (DMF) and 3.8 mg anhydrous 1-hydroxybenzotriazole (HOBt). To that were added 3.5 mg N'N-diisopropylcarbodiimide (DIC) and the mixture was agitated at room temperature for 0.5 h. Then 15 mg of BMS-α were dissolved in 2 mL DMF, and stirred at room temperature for 24 h. Finally, the target product (DSPE-PEG-BMS-α) was obtained by dehydration synthesis of BMS-α and DSPE-PEG-COOH, which identified and purified by high-performance liquid chromatography (HPLC).

The glucocorticoid nano-delivery system was synthesized by a lipid thin film and a simple facile acoustic-emulsification method. First, a confirmed molar ratio (DPPC:DPPG:DSPE-PEG-BMS-α:Chol = 69:8:8:15) of 10 mg hybrid lipids and 4 mg Dex were dissolved in a mixture of 5 mL trichloromethane (CHCl_3_) and 2 mL methyl alcohol (CH_3_OH). The mixed solution was transferred into a 100 mL round-bottomed flask to form the fused lipid membrane through continuous rotary evaporation in a water bath at 52 °C for 1 h. Next, 2 mL PBS was added to fully hydrate the fused lipid membrane and the mixture was transferred to a 10 mL PE tube in an ice bath. Then, 200 μL PFP was added to the mixture. The solution was sonicated using an ultrasonic probe (Sonics & Materials Inc., USA) for 5 s on and 5 s off for a total of 5 min at 100 W in an ice bath. The glucocorticoid nano-delivery system of Dex/PFP@LIPs-BMS-α was obtained. To purify the Dex/PFP@LIPs-BMS-α, a cryogenic centrifuge washing process was used (4 °C, 8000 rpm, 8 min).

Dex/PFP@LIPs and PFP@LIPs-BMS-α were obtained by the same methods as described above with the exception that the DSPE-PEG-BMS-α was replaced by DSPE-PEG-COOH, and Dex was not added. The preparation of PFP@LIPs was similar to the description above except the raw material included only DPPC, DPPG, DSPE-PEG-COOH and Chol. To obtain the fluorescently labeled glucocorticoid nano-delivery system, DiI (0.2 mg/10 mg liposomes) or DiR (0.4 mg/10 mg liposomes) were added to the hybrid lipids solutions.

### Characterization of Dex/PFP@LIPs-BMS-α

The morphology and structure of Dex/PFP@LIPs-BMS-α were observed by transmission electron microscope (TEM, Hitachi H-7600, Japan). The size distribution was determined by a laser particle size analyzer system (Nano, ZS90, Malvern instrument Ltd) that detected the size distribution of 7 d and 14 d in PBS or serum. The Zeta potentials of PFP@LIPs, Dex/PFP@LIPs and Dex/PFP@LIPs-BMS-α also were detected by a malvern Zeta sizer. The optical absorption properties of Dex, PFP@LIPs-BMS-α and Dex/PFP@LIPs-BMS-α were assessed by an Ultraviolet-visible (UV-vis) spectrophotometer (UV-3600, Shimadzu, Japan), and the standard concentration curves of Dex were drawn according to the spectral values of free Dex at 242 nm with different concentrations by UV-vis and HPLC with C18 column (4.6 × 250 mm, 5 μm). Acetonitrile and water (V/V = 4:6) were used as mobile phase, and the column temperature was set as 25 °C. Dex/PFP@LIPs-BMS-α was centrifuged for 3 times, then the concentration of Dex was determined by UV-vis and HPLC after DMSO dissolution and centrifugation. Accordingly, the encapsulation Efficiency (EE%) and loading efficiency (LE%) of Dex in the glucocorticoid nano-delivery system were calculated according to the following formula: EE% = Total mass of Dex in the glucocorticoid nano-delivery system/Total mass of Dex × 100%; LE% = Total mass of Dex in the glucocorticoid nano-delivery system/Mass of total glucocorticoid nano-delivery system × 100%.

### *In vitro* liquid-gas phase transition, ultrasound imaging and Dex release using LIFU

The glucocorticoid nano-delivery systems were added into preformed wells of agar gel phantom (3% W/V in distilled water). Then, LIFU (1 MHz, duty cycle of 50%, Chongqing Medical University, P.R. China) was used to study the *in vitro* liquid-gas phase transition capability of the glucocorticoid nano-delivery system. We studied the characteristics of liquid-gas phase transition capacity which were time (1-5 min), and acoustic strength dependent (0.8, 1.6 and 2.4 W/cm^2^). Ultrasonic harmonic imaging signals of the glucocorticoid nano-delivery system by LIFU were observed and collected by an ultrasound diagnostic system (Mylab 90, Esaote, Italy). The signal intensity analysis of B-mode and contrast enhanced ultrasound mode (CEUS-mode) was completed by DFY software (Chongqing Medical University, P.R. China). In addition, the liquid-gas phase transition process of glucocorticoid nano-delivery system was recorded by optical microscope.

The optimal parameters for liquid-gas phase transition of glucocorticoid nano-delivery system triggered by LIFU were used in the Dex release study. First, tween-80 (2%, V/V) was added to PBS (pH = 7.4) and configured with 150 mL buffer. Then, for the LIFU group, 4.68 mg Dex/PFP@LIPs-BMS-α was suspended in 2 mL pre-configured buffer. The suspended glucocorticoid nano-delivery system was treated with LIFU (1 MHz, 2.4 W/cm^2^, 3 min, duty cycle of 50%, Chongqing Medical University, P.R. China) before being injected into a dialysis bag (MWCO = 3 k Da). The dialysis bag was incubated in a conical flask with 60 mL pre-configured buffer, pre-warmed at 37 °C, and uninterrupted stirring (120 rpm). To 1 mL release medium was added 1 mL pre-configured buffer at predetermined points in time (0, 2, 4, 8, 16, 24, 48 and 72 h). For the control group, an equivalent amount to the LIFU group was suspended in 2 mL pre-configured buffer and placed directly into dialysis bag (MWCO = 3 kDa). Subsequent experimental schemes were performed in exactly the same manner as the LIFU group.

The Dex concentration in the releasing medium was measured three times by an UV-vis spectrophotometer. The accumulative release of Dex was calculated according to the following equation. *60 (mL)* represents total volume of releasing medium; *1 (mL)* represents the sampling volume; *C_n_* represents concentration of Dex in the sample collected at *n* time point; *M_(Dex)_* represents the total mass of Dex contained in the glucocorticoid nano-delivery system added to the dialysis bag.

Cumulative release (%) = (60 × C_n_ + 1×∑n-1 i=1C_i_)/M_(Dex)_

### Cell culture

Human kidney podocytes from Beina culture collection (Beijing, China) were used in cellular experiments. Human podocytes were cultured in McCoy's 5a medium, supplemented with 10% fetal bovine serum (FBS) and 1% penicillin-streptomycin at 37 °C with 5% CO_2_.

### Cytotoxicity and apoptosis assay

Human kidney podocytes were allowed to differentiate for 13 days at 37 °C in McCoy's 5a medium with 10% FBS and 1% penicillin-streptomycin with 5% CO_2_. Then, cells were seeded in 96-well plates at 1 × 10^4^ cells per well and continued to be cultured for 24 h at 37 °C, 5% CO_2_. Included in the experiment to investigate cytotoxicity were 0.256 mg/mL Dex or various components of the glucocorticoid nano-delivery system (0.483 mg/mL PFP@LIPs, 0.739 mg/mL Dex/PFP@LIPs, 0.544 mg/mL PFP@LIPs-BMS-α and 0.8 mg/mL Dex/PFP@LIPs-BMS-α). Similarly, concentrations of Dex (0, 0.032, 0.064, 0.128, 0.192, 0.256 and 0.32 mg/mL with fresh medium), PFP@LIPs-BMS-α (0, 0.068, 0.136, 0.272, 0.408, 0.544 and 0.68 mg/mL with fresh medium) and Dex/PFP@LIPs-BMS-α (0, 0.1, 0.2, 0.4, 0.6, 0.8 and 1 mg/mL with fresh medium) were included in the experiment to investigate cytotoxicity. The dosage of the three experimental variables was analogous. First, fresh medium containing various experimental variables were added to 96-well plates and replaced with 10 μL CCK-8 solution and 190 μL serum-free medium to each-well at the end of incubation time (24 h). The plates were incubated at 37 °C, 5% CO_2_ for another 4 h. The absorbance was measured at OD = 460 nm by microplate spectrophotometer system. In addition, nanoparticles may interfere with commonly used toxicity test systems 42. Therefore, we evaluated the apoptotic proteins of Bax and Bcl-2 in cells by western blot (WB).

### Western blot

Briefly, podocytes were incubated with 0.256 mg/mL Dex or various components of the glucocorticoid nano-delivery system (0.483 mg/mL PFP@LIPs, 0.739 mg/mL Dex/PFP@LIPs, 0.544 mg/mL PFP@LIPs-BMS-α and 0.8 mg/mL Dex/PFP@LIPs-BMS-α) as described for the cytotoxicity assay after PA induction. Protein lysates of cells were prepared in accordance with standard procedures, and protein concentrations were determined using a BCA protein assay kit (Thermoscientific). Then, equivalent protein samples were separated by electrophoresis under reduced conditions, and transferred to the polyvinylidene difluoride (PVDF) membranes in accordance with standard procedures. Membranes were blocked with 5% nonfat dry milk in tris-buffered saline-Tween 20 for 1 h at room temperature and were incubated with primary antibodies overnight at 4 °C. The following primary antibodies were used: anti-MC1R antibody (ab236734, ab180776, abcam), anti-Bax antibody (ab32503, abcam), anti-Bcl-2 antibody (ab59348, abcam). Then, membranes were incubated with anti-rabbit horseradish peroxidase-conjugated antibodies for 2 h at room temperature after washing, and the immunoreactive bands were detected by enhanced chemiluminescence using an ECL system, β-actin was used as the reference of relative protein expression intensity.

### Targeted receptor confirmation and expression *in vitro*

To study receptor expression of targeted binding on the surface of human kidney podocytes, which were allowed to differentiate for 8 days at 37 °C in McCoy's 5a medium with 10% FBS and 1% penicillin-streptomycin with 5% CO_2_. Then, cells received various treatments including fresh medium (control), or fresh medium with the following: PA (5 μg/mL), PA (5 μg/mL) + BMS-α (10 nM) and PA (5 μg/mL) + DSPE-PEG-BMS-α (with 10 nM BMS-α) for 72 h at 37 °C with 5% CO_2_. After treatment, cells were inoculated in a confocal dish and cultured in fresh medium for another 24 h until the right degree of cell fusion was achieved. PBS containing 4% paraformaldehyde was then used to immobilize the cells for 15 min at room temperature and subsequently washed thrice with PBS. After immobilization, cells were permeabilized with PBS containing 0.1% triton X-100 (Macklin Biochemical Co., Ltd. Shanghai, China) at room temperature for 5 min and washed thrice with PBS. Cells were then blocked with PBS containing 1% BSA (Macklin Biochemical Co., Ltd. Shanghai, China) for 1 h and washed again. Subsequently, to cells were added 100 μL anti-MC1R antibody (MC-1R, ab236734, abcam) at a dilution of 1:130 and left overnight at 4 °C. Then, to cells were added 100 μL goat anti-rabbit IgG H&L (Alexa Fluor® 488) at a dilution of 1:1000 for 1 h at room temperature, then washed three times. The nuclei were stained with DAPI for 10 min and washed thrice again with PBS. Finally, fluorescence imaging was observed and recorded by confocal laser scanning microscopy (CLSM, Nikon A1R-si, Tokyo, Japan). In the same scheme above, the MC-1R expression in human kidney podocytes was verified according to WB. In addition, the expression of MC-1R in PHN and Normal rat's organs including heart, liver, spleen, lung, kidney, cerebrum, skin and testis were verified by immunofluorescent staining (PA5-75342, Thermo Fisher) and WB (ab180776, abcam).

### Cell targeting efficiency and flow cytometry

To study the targeting ability of Dex/PFP@LIPs-BMS-α *in vitro*, the experiment consisted of a target free group (Dex/PFP@LIPs) and a target group (Dex/PFP@LIPs-BMS-α), all marked by DiI. First, human kidney podocytes were inoculated in confocal culture dishes and cultured to a suitable degree of cell fusion at 37 °C, 5% CO_2_ in a humidified incubator. The target free group was co-incubated with 40 μL Dex/PFP@LIPs (2.5 mg/mL) including fresh medium for either 30, 60 or 120 min at 37 °C with 5% CO_2_. The target group was co-incubated with 40 μL Dex/PFP@LIPs-BMS-α (with 2.5 mg/mL Dex/PFP@LIPs) including fresh medium under the same condition as the target free group. After co-incubation, PBS containing 4% paraformaldehyde immobilized the cells for 15 min at room temperature, then washed thrice with PBS. The nuclei were stained with DAPI for 10 min and washed thrice again with PBS. Finally, fluorescence imaging was observed and recorded by CLSM.

Flow cytometry was performed to further evaluate the cell targeting efficiency. The cells were grouped according to the above-described experimental protocol and inoculated into 6-well plates for 24 h at 37 °C, 5% CO_2_ in a humidified incubator. After co-incubation, washing with PBS, digestion and centrifugation, the cells were collected and washed again with PBS. Finally, the amount of targeted binding was analyzed by flow cytometry.

### Determination of Reactive Oxygen Species (ROS) levels

In order to investigate whether Dex/PFP@LIPs-BMS-α combined with or without LIFU (1 MHz, 2.4 W/cm^2^, 3 min, duty cycle of 50%) could reduce the expression level of ROS in podocytes. Podocytes were allowed to differentiate for 8 days at 37 °C in McCoy's 5a medium with 10% FBS and 1% penicillin-streptomycin with 5% CO_2_. Then, cells received various treatments of fresh medium only (control), Dex (0.256 mg/mL), PFP@LIPs-BMS-α (0.544 mg/mL), Dex/PFP@LIPs-BMS-α (0.8 mg/mL), Control + LIFU, Dex (0.256 mg/mL) + LIFU, PFP@LIPs-BMS-α (0.544 mg/mL) + LIFU, Dex/PFP@LIPs-BMS-α (0.8 mg/mL) + LIFU for 24 h at 37 °C with 5% CO_2_ after PA induction (72 h). Of course, the cells were inoculated into the confocal dish before treatment began. Then, 25 μM DCFDA was added and allowed to incubate for 45 min after washed twice with buffer. In addition, those treated with tert-butyl hydrogen peroxide were designated as the positive control group. ROS development was observed by CLSM (λ_ex_/λ_em_ = 485 nm/535 nm) after washed again with buffer. The fluorescence intensity was analyzed by a semi-quantitative method.

### Animal and model

All animal experiments were performed on male Sprague-Dawley (SD) rats with initial body weight of 130 g to 170 g, as approved by the animal ethics committee of Chongqing Medical University. All animals were provided and raised by the laboratory animal center in Chongqing Medical University.

To induce passive heymann nephritis (PHN), 1.5 mL anti-Fx1A was slowly injected into the tail vein at day 0, and 0.5 mL was injected again on the 7^th^ day. In order to evaluate PHN, urine was collected weekly to measure proteinuria and body weight was recorded weekly from the first injection of antibodies until the 14^th^ day. On the 14^th^ day, the morphology of renal podocytes and glomerular basement membrane were observed under TEM (Hitachi H-7600, Japan).

### Kidney targeting efficiency and biodistribution

Photoacoustic imaging (PAI) and fluorescent imaging were used to evaluate kidney targeting efficiency and biodistribution.

For *in vivo* PAI, first, DiR-labeled glucocorticoid nano-delivery systems were scanned from 680 nm to 970 nm, using a PAI system (Visual Sonics Inc., Toronto, Canada), to determine maximum absorbance. Then, a laser emitter with an excitation wavelength of 770 nm was used. The *in vitro* PAI and photoacoustic values of Dex/PFP@LIPs-BMS-α (per 10mg) were determined at different DiR concentrations (25, 50, 100, 200, 300, 400 and 500 μg). PHN rats were injected through a tail vein with Dex/PFP@LIPs (2.5 mg/mL, 500 μL, labeled by DiR) or Dex/PFP@LIPs-BMS-α (with 2.5 mg/mL Dex/PFP@LIPs, 500 μL, labeled by DiR), dissolved in saline. Finally, photoacoustic images and the average photoacoustic-signal intensity were observed and measured at various intervals (Pre, 4, 8, 16 and 24 h) after the injection. Vevo LAZR software was used to measure the photoacoustic-signal intensity.

For *in vivo* fluorescent imaging, PHN rats were injected through a tail vein with Dex/PFP@LIPs (2.5 mg/mL, 500 μL, labeled by DiI) or Dex/PFP@LIPs-BMS-α (with 2.5 mg/mL Dex/PFP@LIPs, 500 μL, labeled by DiI), dissolved in saline. Then, the PHN rats were anesthetized at predetermined time points (pre, 4, 8, 16, 24 and 48 h), the ipsilateral kidney was surgically removed. Frozen section kidney specimens were prepared and the nuclei were stained with DAPI. Finally, Fluorescence imaging was performed by CLSM at λ_ex_/λ_em_ = 488 nm/525 nm, λ_ex_/λ_em_ = 544 nm/595 nm and λ_ex_/λ_em_ = 400 nm/450 nm. Additionally, the PHN rats were anesthetized and the heart, liver, spleen and lung were surgically removed at 48 h. After rapid freezing, ultrathin sections were prepared and the nuclei stained by DAPI. Finally, fluorescence imaging of biodistribution was performed by CLSM at λ_ex_/λ_em_ = 544 nm/595 nm, λ_ex_/λ_em_ = 364 nm/454 nm. The fluorescence intensity was analyzed by semi-quantitative method.

### Restoration of stress fibers after injury in podocytes

To study damaged stress fibers of podocytes by targeting ligands. Human kidney podocytes were allowed to differentiate for 7 days at 37 °C in McCoy's 5a medium with 10% FBS and 1% penicillin-streptomycin with 5% CO_2_. Then, cells received various treatments of fresh medium only (control), PA (5 μg/mL), PA (5 μg/mL) + Dex (0.544 mg/mL), PA (5 μg/mL) + BMS-α (100 nM) and PA (5 μg/mL) + DSPE-PEG-BMS-α (with 100 nM BMS-α) for 72 h at 37 °C with 5% CO_2_. After treatment, cells were inoculated in a confocal dish and cultured in fresh medium for another 24 h. Then, PBS containing 4% paraformaldehyde immobilized the cells for 15 min at room temperature and was subsequently washed thrice with PBS. After immobilization, cells were permeabilized with PBS containing 0.1% triton X-100 at room temperature for 30 min, and washed thrice with PBS. Cells were then blocked with PBS containing 1% BSA for 15 min and washed thrice again. Subsequently, cells were added to TRITC-phalloidin (100 nM, 300 μL) for 30 min at room temperature. The nuclei were stained with DAPI for 10 min and washed thrice again with PBS. Finally, fluorescence imaging was observed and recorded by CLSM (Nikon A1R-si, Tokyo, Japan) at λ_ex_/λ_em_ = 544 nm/595 nm, λ_ex_/λ_em_ = 364 nm/454 nm. The fluorescence intensity was analyzed by a semi-quantitative method. The state of the stress fiber was evaluated by semi-quantitative fluorescence analysis.

### Efficacy and side-effects *in vivo*

As mentioned above, PHN model rats were prepared by the above protocol and divided into eight groups including control (n = 5), Dex (n = 5), PFP@LIPs-BMS-α (n = 5), Dex/PFP@LIPs-BMS-α (n = 5), Control + LIFU (n = 5), Dex + LIFU (n = 5), PFP@LIPs-BMS-α + LIFU (n = 5), and Dex/PFP@LIPs-BMS-α + LIFU (n = 5). Rats with proteinuria quantified greater than 160 mg/24 h at 14^th^ after injection of anti-Fx1A were included in the follow-up treatment study. The therapeutic agents (3.96 mg/kg of DSP, containing 3 mg/kg of Dex. 6.36 mg/kg of PFP@LIPs-BMS-α; or 9.36 mg/kg of Dex/PFP@LIPs-BMS-α) were injected into the tail vein of PHN rats every three days and treated twice with LIFU (1 MHz, 2.4 W/cm^2^, 3 min, duty cycle of 50%) at 8 h and 16 h after injection. All treatments continued for 4 weeks. Rats with severe symptoms of infection, edema, weight loss (less than 170 g), and elevated proteinuria during treatment were sacrificed.

To determine therapeutic efficacy and side-effects, weekly measurements were recorded of body weight, proteinuria, and peripheral blood was obtained for serum IgG, white blood cells (WBC), glutamic-pyruvic transaminase (ALT) and serum creatinine. Proteinuria was measured by urine protein test kit (Nanjing Jiancheng, Inc., Nanjing, China); serum IgG was measured by ELISA using enzyme-linked immunosorbent assay kit for immunoglobulin G (Cloud-clone Inc. Houston, TX, USA); WBC was measured by automatic hematology analyzer (BC-2800 Vet, Mindray Inc., Shenzhen, China); ALT, serum creatinine, blood glucose, total cholesterol and triglyceride were measured by automatic biochemical analyzer (Chemray-240, Rayto Inc., Shenzhen, China).

For histology and immunohistochemistry, hematoxylin-eosin (H&E) staining was performed on the heart, liver, spleen and lung tissues of each group after treatment. The kidneys were fixed with 4% paraformaldehyde solution and embedded in paraffin, and kidney sections were stained with periodic Acid-Schiff (PAS). The degree of glomerular and basement membrane changes by PAS staining was quantitatively evaluated using the ratio: glomerular positive pixel area/glomerular pixel area by Image-pro plus 6.0 (Media Cybernetics, Inc., Rockville, MD, USA). Rabbit anti-CD3 antibody was used for CD3 T-cells staining according to the manufacturer's protocol. The expression level of CD3 T-cells by immunohistochemistry was quantitatively evaluated, by evaluation of the number of CD3 T-cells/field by Image-pro plus 6.0. In addition, kidneys were observed by TEM (Hitachi H-7800, Japan). At least three ipsilateral kidneys of rats were selected from each group and at least 15 μm^2^ were selected for measurement of the foot process width by Image J. To evaluate the therapeutic efficacy and side-effects of treatment, the normal rats and PHN model rats were included in the PAS staining, foot process width and CD3 analysis.

In addition, the relationship between the dexamethasone nano-delivery system and renal metabolism in immune-associated nephropathy was studied by analyzing blood glucose, total cholesterol and triglyceride after Dex/PFP@LIPs-BMS-α treatment.

### Statistical analysis

All data were presented as mean ± standard (mean ± SD) and analyzed by SPSS 24.0 software. Statistical analyses were performed using one-way analysis of variance (one-way ANOVA) of comparison among ≥ 3 groups. The Tukey's post-hoc t-test or Student's t-test was used for the pairwise comparisons. *P <* 0.05 was considered statistically significant.

## Results and Discussion

### Synthesis and characterization of Dex/PFP@LIPs-BMS-α

**Figure 1:**
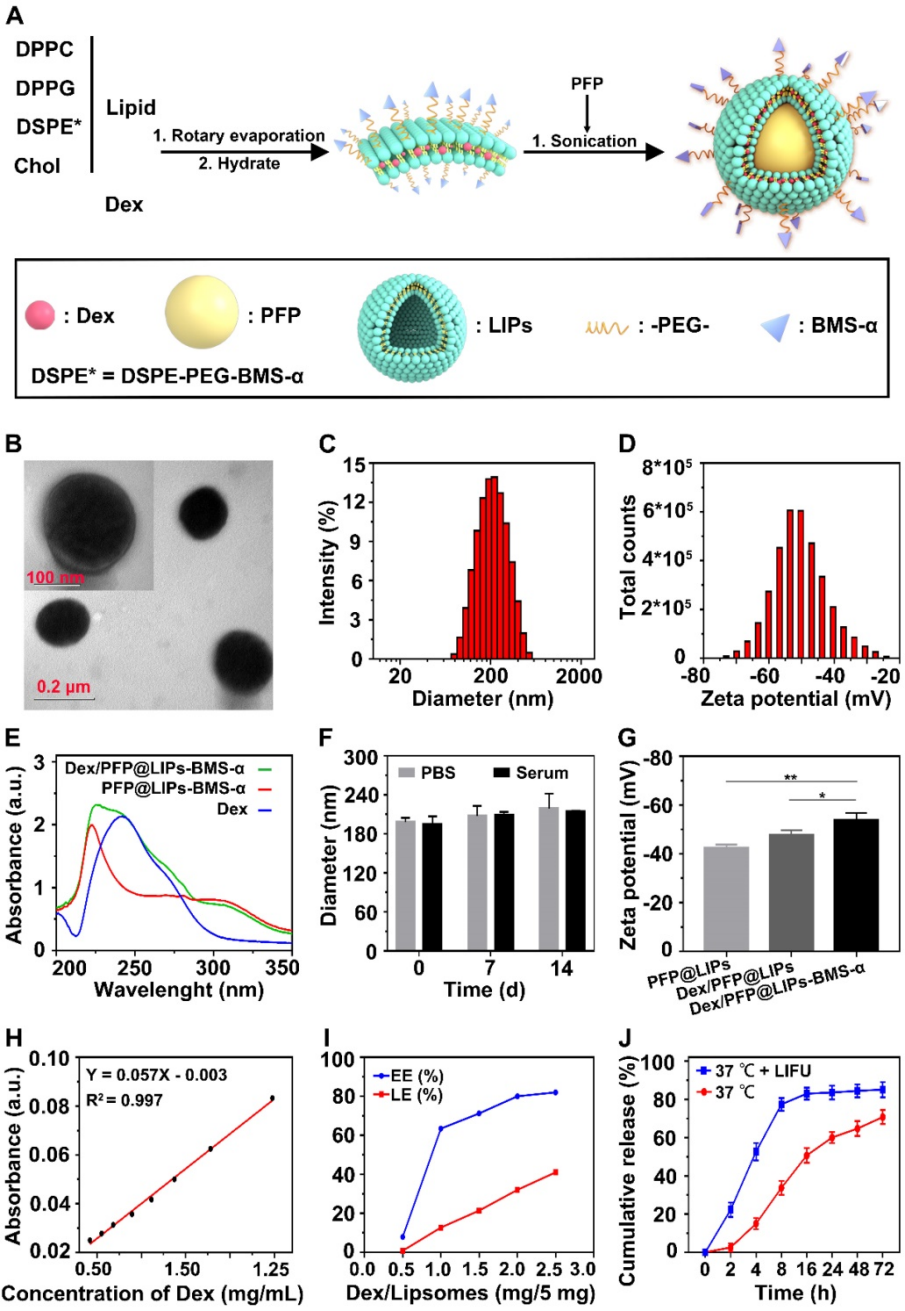
**Synthetic process and characterization of the Dex/PFP@LIPs-BMS-α.** (A) Development agreement of Dex/PFP@LIPs-BMS-α. DPPC: 1,2-dipalmitoyl-sn-glycero-3-phosphatidylcholine, DPPG: 1,2-dipalmitoyl-sn-glycero-3-phospho-(1-rac-glycerol), Chol: cholesterol, PFP: perfluoropentane, Dex: dexamethasone. (B) TEM image of Dex/PFP@LIPs-BMS-α, scale bar = 0.1 μm. (C) The size distribution of Dex/PFP@LIPs-BMS-α. (D) The Zeta potential of Dex/PFP@LIPs-BMS-α. (E) The optical absorption properties of Dex, PFP@LIPs-BMS-α and Dex/PFP@LIPs-BMS-α. (F) The size distribution of Dex/PFP@LIPs-BMS-α at 0^th^, 7^th^ and 14^th^ in PBS and serum (*P <* 0.05). (G) The Zeta potential of PFP@LIPs, Dex/PFP@LIPs and Dex/PFP@LIPs-BMS-α. (H) Standard concentration curve of Dex by UV-vis. (I) The EE% and LE% of Dex. (J) The release rate of Dex under different conditions (LIFU, 1 MHz, 2.4 W/cm^2^, 3 min, duty cycle of 50%). Data are presented as mean ± SD, n = 3, one-way ANOVA, *P < 0.05, **P < 0.01.

DSPE-PEG-BMS-α copolymer was prefabricated by the carbodiimide method prior to synthesis of Dex/PFP@LIPs-BMS-α (**Figure S1**), utilizing HPLC to verify the copolymer (**Figures S2**). These results indicated that BMS-α and DSPE-PEG-COOH were conjugated successfully by carbodiimide method, and reaching a purity of 90.83% by HPLC after verification and purification. The Dex/PFP@LIPs-BMS-α was prepared by a lipid thin film and a simple facile acoustic-emulsification method. A visualized podocyte-targeting and focused ultrasound responsive glucocorticoid nano-delivery system was harvested with BMS-α that was externally connected, and Dex and PFP that were internally loaded (**Figure 1A**). As shown in **Figure 1B**, Dex/PFP@LIPs-BMS-α exhibited a highly singular, dispersible, unfocused and spherical appearance under TEM. The laser particle size analyzer system showed that the particle size distribution of Dex/PFP@LIPs-BMS-α was around 190.14 nm (**Figure 1C**). The Zeta potential measured by Malvern Zeta Sizer was -50.5 ± 8.14 mV for Dex/PFP@LIPs-BMS-α (**Figure 1D**). A negative Zeta potential is conducive to the prevention of glucocorticoid nano-delivery system self-aggregation and protein aggregation with negative potential *in vivo* 43. The results of the UV-vis analysis verified that Dex/PFP@LIPs-BMS-α had a characteristic absorption peak that overlapped with Dex at 242 nm, and a characteristic absorption peak that overlapped with PFP@LIPs-BMS-α at around 223 nm. These qualitative analysis results indicated that Dex was successfully encapsulated (**Figure 1E**). Dex/PFP@LIPs-BMS-α were placed in PBS at 4 °C and in serum at room temperature, and there were no statistically significant (**Figure 1F**) differences in particle size distribution on day 7 and day 14 compared with day 0 (**Figure S3A-B**). The Zeta potential of Dex/PFP@LIPs-BMS-α was higher than that of simple liposomes (PFP@LIPs) and drug-delivery liposomes without ligands (Dex/PFP@LIPs) (**Figure 1G**), indicating that Dex/PFP@LIPs-BMS-α had a high level of stability. **Figure 1H** showed the standard concentration curve of Dex obtained by UV-vis analysis (**Figure S4**), which provided support for obtaining EE% and LE% (**Figure 1I**). Similarly, the HPLC indicated similar results with the Dex EE% and LE% (**Figures S5** and** S6**) as compared to the UV-vis analysis. Different amounts of Dex (0.5, 1.0, 1.5, 2.0 and 2.5 mg) were added to 5 mg liposomes to prepare Dex/PFP@LIPs-BMS-α. As shown in **Figure S6**, the EE% of Dex was 84.95% when 2 mg Dex was added, but it reduced to 80.55% when 2.5 mg Dex was added. We determined that when a dose of 2 mg Dex was used, the EE% achieved the optimal ratio. Therefore, in this study, preparation of Dex/PFP@LIPs-BMS-α utilized this optimal ratio of 2 mg Dex for each 5 mg of liposomes.

### The release rate of Dex

LIFU triggered Dex/PFP@LIPs-BMS-α stripping of Dex was simulated *in vitro* (**Figure 1J**). At 37 °C without LIFU, the Dex release rate at 2 h was only 2.55 ± 2.13%. With LIFU triggering, the Dex release rate at 2 h measured 22.30 ± 3.77%, and the Dex release rate at 8h reached 77.36 ± 3.36%, much higher than without LIFU (33.68 ± 3.67%). These results indicated that LIFU was both feasible and efficient as a tool for inducing drug release, concordant with previously published studies 44. These parameters for LIFU to trigger Dex release were derived from liquid-gas phase transition induced by LIFU *in vitro*.

### Liquid-gas phase transition and ultrasonic imaging by LIFU

**Figure 2:**
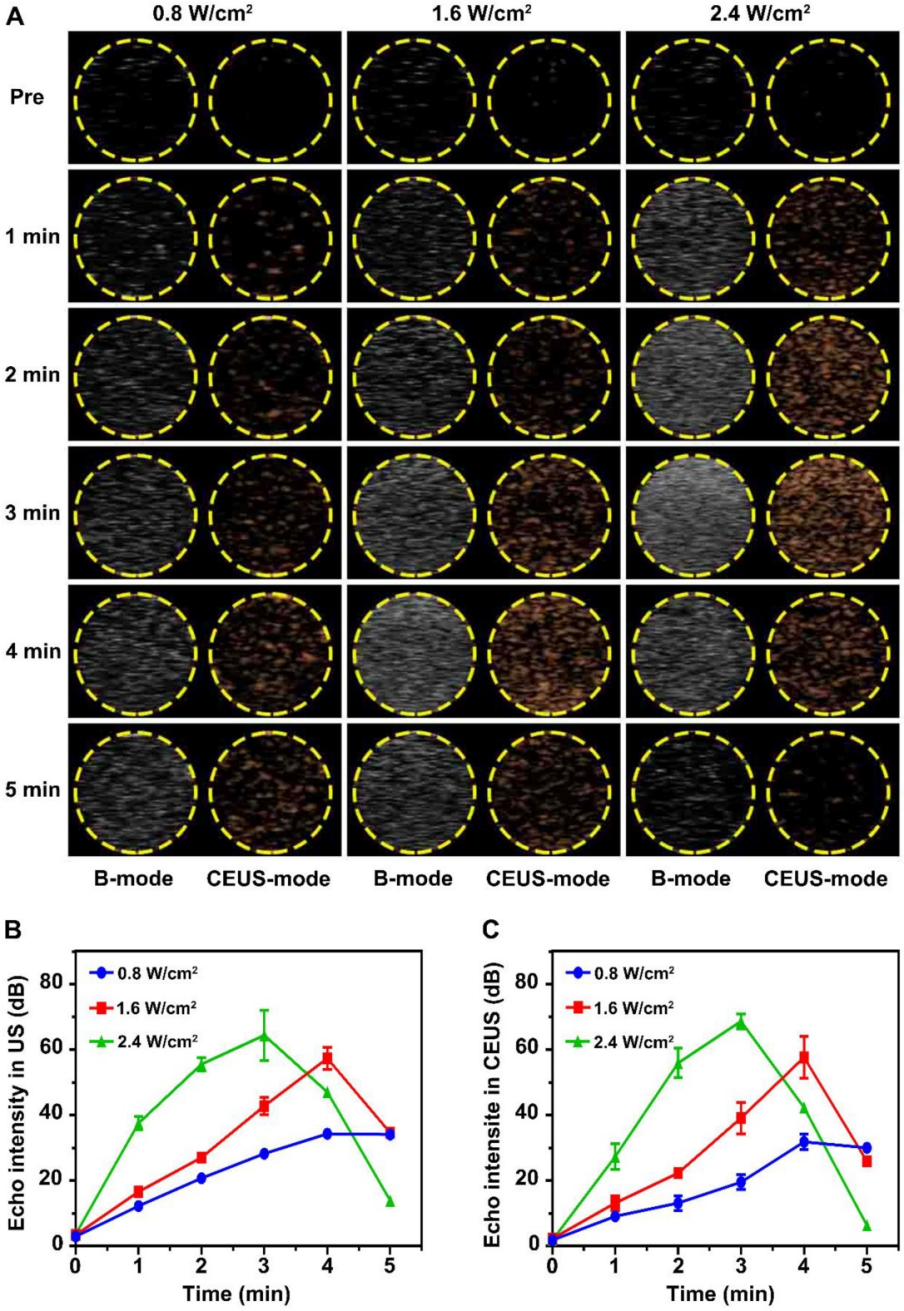
**Liquid-gas phase transition using LIFU, and ultrasound imaging of Dex/PFP@LIPs-BMS-α *in vitro*.** (A) B-mode and CEUS-mode ultrasound imaging of Dex/PFP@LIPs-BMS-α after being irradiated by LIFU at different points in time (pre, 1-5 min) and acoustic strength (0.8, 1.6 and 2.4 W/cm^2^). Quantitative analysis of signal intensity in B-mode (B) and CEUS-mode (C). Data are presented as mean ± SD.

Liquid-gas phase transition and ultrasonic imaging induced by LIFU were simulated in an agar gel phantom. As shown in **Figure 2A**, the imaging capability after LIFU induced ADV effect was time and acoustic strength dependent. Before LIFU action, only an extremely weak echo signal could be detected in ultrasonic B-mode, while almost no echo signal could be detected in CEUS-mode. The echo signal gradually increased with the action time up 5 min under the intensity of 0.8 W/cm^2^. The strongest echo signal was detected after 4 min at the strength of 1.6 W/cm^2^, but began to attenuate slowly at 5 min. The echo signal was strongest after 3 min at 2.4 W/cm^2^ intensity, and then decayed rapidly. **Figure 2B**-**C** depicts the variation curves of signal strength in B-mode and CEUS-mode. In addition, the liquid-gas phase transition process of glucocorticoid nano-delivery system was recorded by optical microscope (**Figure S7**). These demonstrated that LIFU could trigger ADV effect and achieve the visualization ultrasonic monitoring of the Dex/PFP@LIPs-BMS-α. Therefore, the implementation of UTMD by LIFU was verified. Noteworthy, we verified that the parameters of UTMD were different from previous studies on nanoliposomes 45,46. Study showed that the molar ratio of lipids played a key role in defining final liposome characteristics 47. Briuglia ML *et al.* studied different lipid ratios to prepare stable and controlled drug nano-delivery vectors 48, which were evaluated by stability. Li Y *et al.* studied the relationship between shell hardness and the transmission efficiency of the drug nano-delivery system, as well as considering stability 49. As in our study, adding cholesterol in lipids was helpful to improve the stability and the membrane hardening effect of the dexamethasone nano-delivery system 50. Therefore, we considered that the parameters of liquid-gas phase transition by LIFU in Dex/PFP@LIPs-BMS-α may be related to shell hardness, which was determined by lipid ratio.

### Cytotoxicity and apoptosis assay of Dex/PFP@LIPs-BMS-α

**Figure 3:**
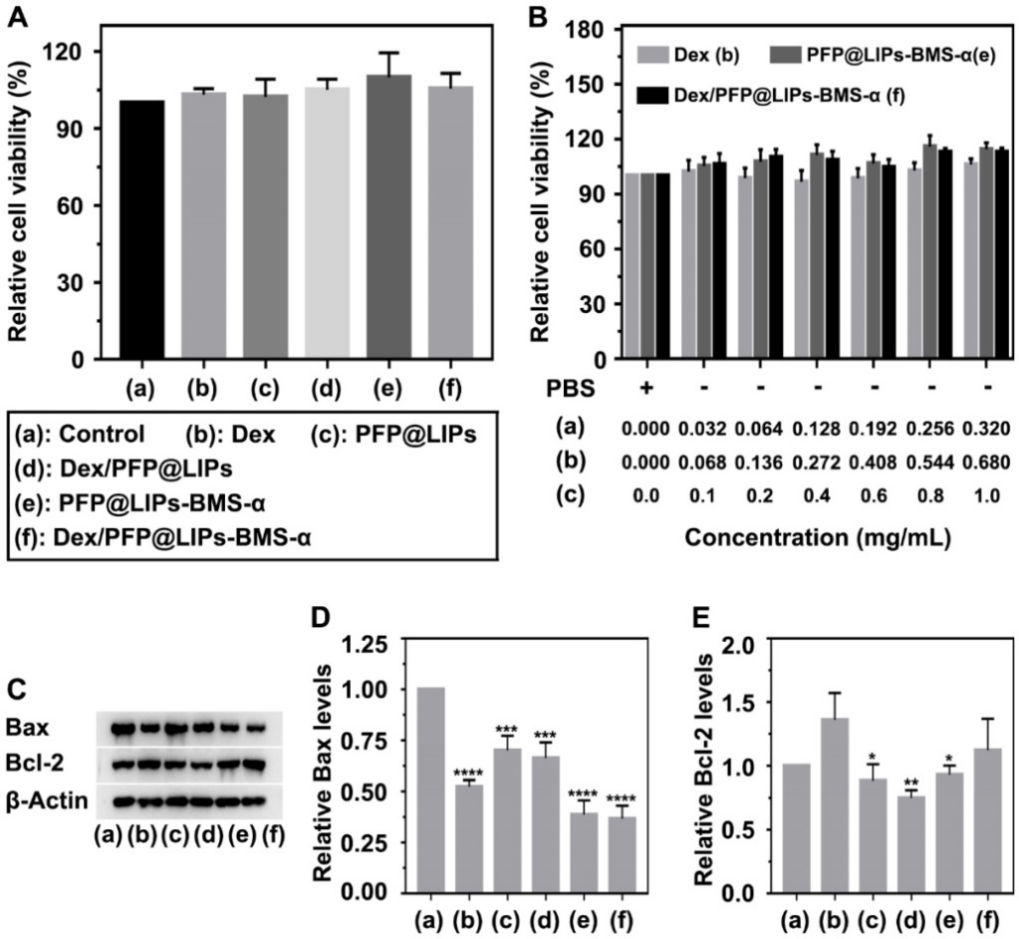
**Cytotoxicity and apoptosis assay of Dex/PFP@LIPs-BMS-α *in vitro*.** (A) Relative cell viability of human kidney podocytes after co-incubation 24 h with Dex and various components of Dex/PFP@LIPs-BMS-α, the dosages of the experimental variable were analogous. (B) Relative cell viability of human kidney podocytes after co-incubation 24 h with different concentrations of Dex, PFP@LIPs-BMS-α and Dex/PFP@LIPs-BMS-α. WB analysis of Bax and Bcl-2 (C-E). Data are presented as mean ± SD, one-way ANOVA, *P < 0.05, ****P < 0.0001.

A CCK-8 kit was used to determine the cytotoxicity of Dex/PFP@LIPs-BMS-α on human kidney podocytes (**Figure 3A**-**B**). First, we investigated the dose-dependent cytotoxicity of Dex, PFP@LIPs-BMS-α and Dex/PFP@LIPs-BMS-α. The results showed that there was no significant cytotoxicity with increasing concentrations. Subsequently, no significant cytotoxic effect was found in the podocytes after co-incubation with various components of the Dex/PFP@LIPs-BMS-α. Apoptin analysis of Bax and Bcl-2 by WB (**Figure 3C-E**) further confirmed that Dex/PFP@LIPs-BMS-α had no cytotoxicity and promoted cell proliferation, liposomes have been approved for clinical use as a safe and non-toxic material 51. In addition, these results also indicated that BMS-α combined with MC-1R could inhibit apoptosis by stabilizing and repairing the podocyte actin cytoskeleton.

### Targeted receptor confirmation and expression *in vitro*

**Figure 4:**
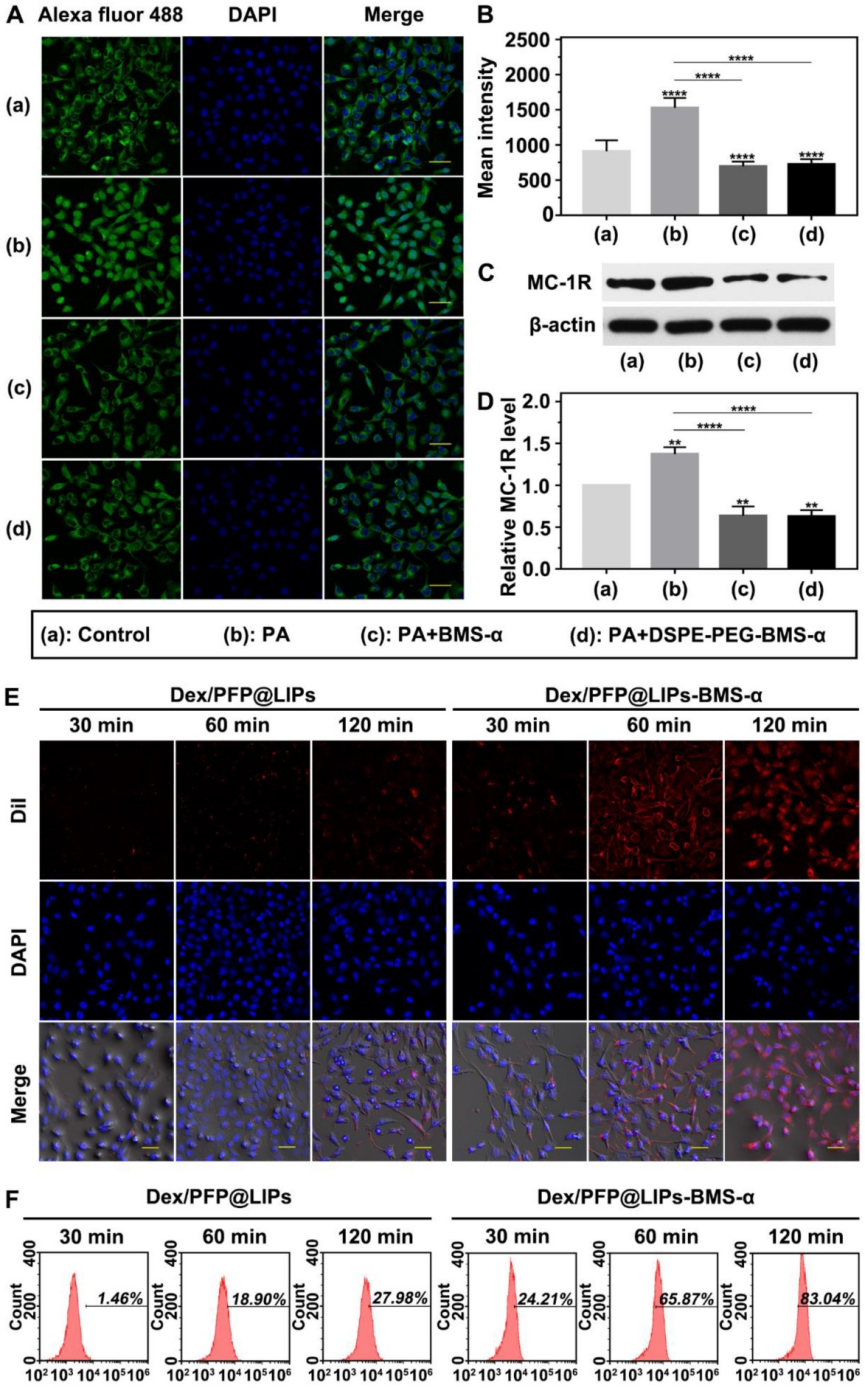
** Targeted receptor confirmation and cell targeting efficiency of Dex/PFP@LIPs-BMS-α *in vitro*.** (A) Targeted receptor of human kidney podocytes were observed by CLSM after different treatment, including control, PA, PA + BMS-α and PA + DSPE-PEG-BMS-α. The excitation and observation of fluorescence were realized through goat anti-rabbit IgG H&L (Alexa Fluor® 488) (green). The nuclei were stained blue, scale bar = 50 µm. (B) The mean fluorescence intensities were measured by a semi-quantitative method. (C and D) The expression levels of MC-1R were analysis by WB. (E) *In vitro* targeting efficiency of Dex/PFP@LIPs-BMS-α were observed by CLSM after different co-incubation times (30 min, 60 min and 120 min). Dex/PFP@LIPs (as a comparison) and Dex/PFP@LIPs-BMS-α were pre-labeled by DiI (red), scale bar = 20 µm. (F) Flow cytometry determined the cell targeting efficiency of Dex/PFP@LIPs and Dex/PFP@LIPs-BMS-α at the same point in time. Data are presented as mean ± SD, one-way ANOVA, **P < 0.01,****P < 0.0001, vs. control group.

The expression levels of MC-1R in podocytes are a prerequisite for specific targeting of Dex/PFP@LIPs-BMS-α. Studies have shown expression of MC-1R in the podocytes of normal people, nephritis patients and PHN rats 21. **Figure 4A** showed this expression of MC-1R in human kidney podocytes. Circular green fluorescence was observed in the control group indicating MC-1R expression in normal human kidney podocytes. In the PA induced podocyte damage model, green fluorescence was significantly increased compared to the control group (**Figure 4B**), indicating that MC-1R expression increased following podocyte damage, which was consistent with previous research 23,46. Moreover, annular low-intensity green fluorescence was observed following treatment with PA combined with BMS-α and PA combined with DSPE-PEG-BMS-α co-incubation. These results indicated that BMS-α could protect against injury to podocytes by PA, and the condensation of BMS-α with lipid did not change the protective effect of BMS-α on podocytes. Similarly, the expression levels of MC-1R through WB (**Figure 4C-D**) also verified these results.

In addition, as shown in **Figure S8**, statistical results showed no statistically significant difference in MC-1R expression among different tissues in normal SD rat. However, in the PHN model rat, the expression levels of MC-1R in tissues mentioned above were no significantly different from that of in normal SD rat except for that in the renal tissue. Noteworthy, the expression levels of MC-1R in kidney of PHN model rat were significantly higher than that in normal SD rat, which was caused by the increased expression of MC-1R during renal inflammation 23. These results suggested that MC-1R was not only expressed in the kidney. However, the expression levels of MC-1R were significantly increased when renal inflammation occurred. As aforesaid, several studies have shown that only MC-1R was found in the kidney and mainly expressed in podocytes. Therefore, the dexamethasone nano-delivery system had relatively specific podocytes targeting function for immune-associated nephropathy.

### Targeting efficiency and biodistribution

We verified the targeting ability of Dex/PFP@LIPs-BMS-α using human kidney podocytes. Speckled distribution of red fluorescence was observed on the surface of podocyte membranes after co-incubation with DiI-labeled Dex/PFP@LIPs for 120 min (**Figure 4E**). The percentage of fluorescent stained cells was only 27.98% by flow cytometry (**Figure 4F**). However, 75.87% of fluorescently stained cells and circular distribution of red fluorescence were observed on the surface of podocyte membranes after co-incubation with DiI-labeled Dex/PFP@LIPs-BMS-α for 60 min. Even higher intensity red fluorescence (83.04% of fluorescently stained cells) was observed in the cytoplasm after co-incubation for 120 min. These results conclusively indicated active and efficient target recognition of podocytes by BMS-α endowed Dex with specific target ability.

**Figure 5:**
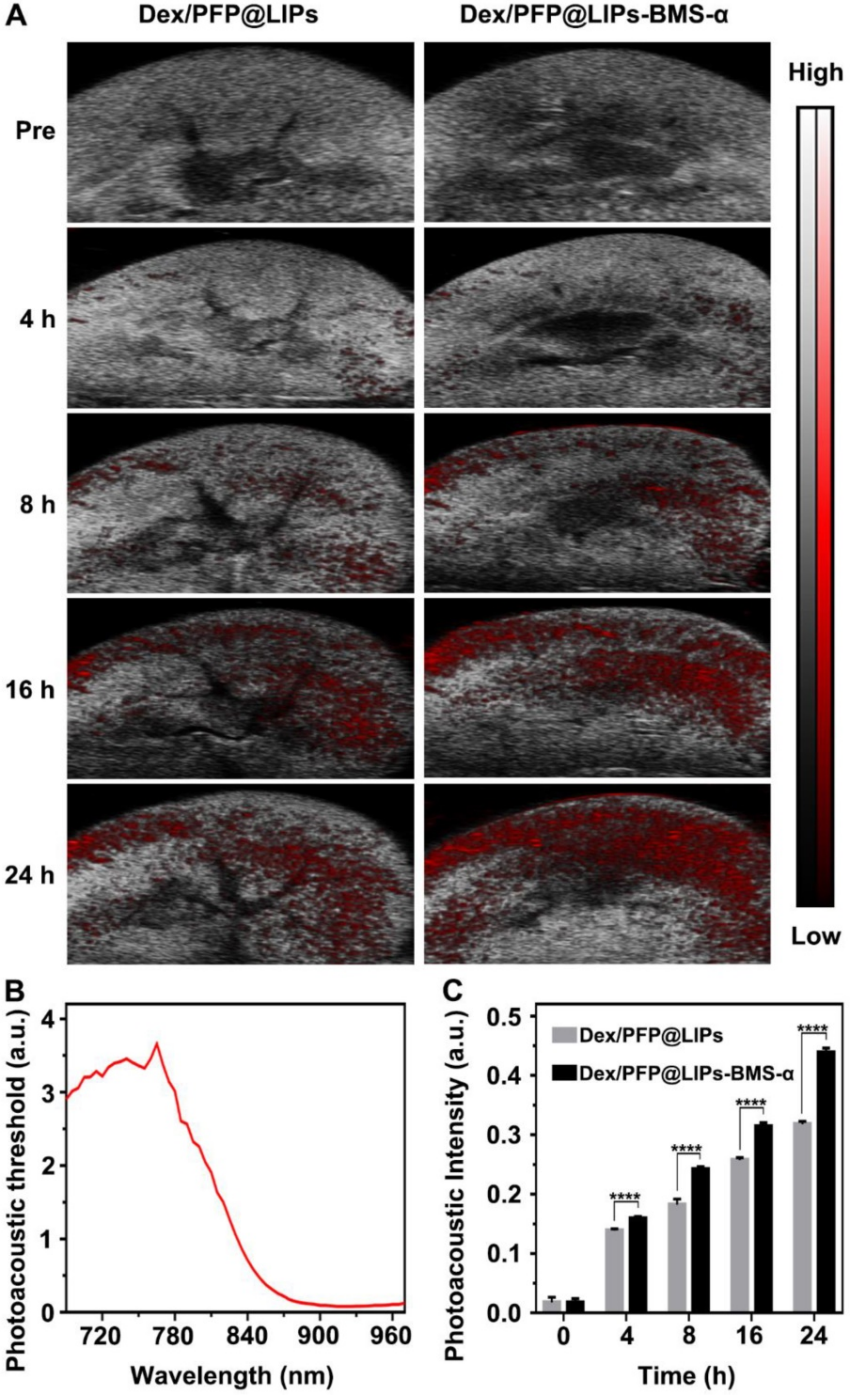
** Kidney targeting efficiency of Dex/PFP@LIPs-BMS-α by organ localization *in vivo*.** (A) *In vivo* targeting efficiency of Dex/PFP@LIPs-BMS-α by organ localization at various intervals (Pre, 4, 8, 16 and 24 h). The photoacoustic signals were shown in red. (B) The photoacoustic threshold of DiR-labeled glucocorticoid nano-delivery system was acquired by PAI system (680-970 nm). (C) Photoacoustic intensities in renal tissue were measured by a Vevo LAZR software at the corresponding point in time. Data are presented as mean ± SD, n = 5, t-test, ****P < 0.0001.

Further, we verified the targeting ability and efficiency of Dex/PFP@LIPs-BMS-α in PHN model rats (**Figure 5A**). The PAI system combining laser and ultrasonic detection has the capability to obtain high resolution optical contrast images of deep tissue 53,54. DiR, a photosensitizer, was used to label the Dex/PFP@LIPs-BMS-α to evaluate the renal targeting capacity through the PAI system. The DiR dose-dependent photoacoustic intensity curve was shown in **Figure S9**. First, we completed an all-band scan (680-960 nm) to determine the optimal excitation wavelength (770 nm) (**Figure 5B**). Weak photoacoustic signals were detected at 8 h after injection of Dex/PFP@LIPs, and slowly increased at 16 h and 24 h. Significant photoacoustic signals were detected at 8 h after Dex/PFP@LIPs-BMS-α injection, which were significantly enhanced at 16 h, and lasted up to 24 h. Photoacoustic intensity results by Vevo LAZR software showed that the Dex/PFP@LIPs-BMS-α and Dex/PFP@LIPs had begun to differ and gradually amplified after 4 h (**Figure 5C**). All of these results indicated that Dex/PFP@LIPs-BMS-α could efficiently target the kidney of PHN model rats.

**Figure 6:**
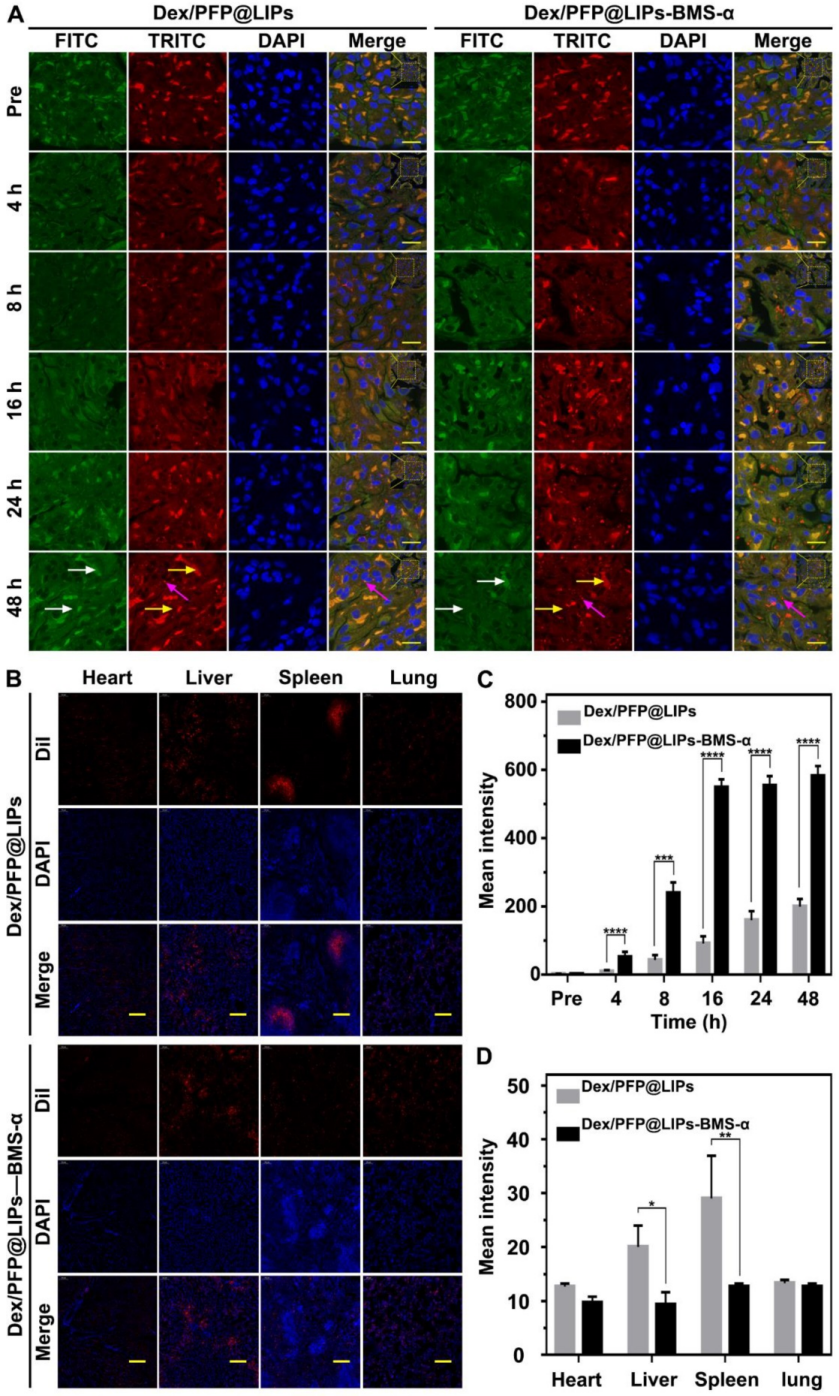
** Kidney targeting efficiency and biodistribution of Dex/PFP@LIPs-BMS-α by cells localization *in vivo*.** (A) *In vivo* efficiency of Dex/PFP@LIPs-BMS-α by cells localization at various intervals (Pre, 4, 8, 16, 24 and 48 h). Green (white arrows) and flaky red (yellow arrows) were self-luminous, and which merged into yellow, the red dots (purple arrows) were the Dex/PFP@LIPs or Dex/PFP@LIPs-BMS-α (labeled by DiI), scale bar = 15 µm. (B) Biodistribution (heart, liver, spleen and lung) in PHN rats were observed by CLSM after injection of Dex/PFP@LIPs or Dex/PFP@LIPs-BMS-α. Dex/PFP@LIPs and Dex/PFP@LIPs-BMS-α were pre-labeled by DiI (red), scale bar = 200 µm. (C) The mean fluorescence intensities were measured by a semi-quantitative method. (D) The mean fluorescence intensities of each groups were measured by Image J. Data are presented as mean ± SD, n = 3, t-test, *P < 0.05, ****P < 0.0001.

We further confirmed the cellular localization and biodistribution of Dex/PFP@LIPS-BMS-α in the kidney. DiI-labeled Dex/PFP@LIPs and Dex/PFP@LIPs-BMS-α were injected into PHN model rats, and kidney tissues were obtained after frozen sections were prepared for study using CLSM. Because tissues have auto-luminescence, we added the FITC channel (green, shown by the white arrow) in our work. Therefore, red auto-luminescence (yellow arrow) and red DiI fluorescence (red) enabled be captured in the TRITC channel. The FITC channel (green) and the TRITC channel (red) could filter out the auto-luminescence after they are merged, and we could trace the glucocorticoid nano-delivery system labeled by DiI in the merged images. The results showed that speckled distribution of red DiI fluorescence signals were observed in the glomeruli of PHN model rats after Dex/PFP@LIPs treatment for 4 h and 8 h (**Figures 6A** and** S10**). Semi-quantitative analysis showed that the red DiI fluorescence signal increased extremely slowly until 48 h (**Figure 6C**). However, DiI fluorescence signals were increased in glomeruli of PHN model rats after Dex/PFP@LIPs-BMS-α treatment for 8 h, and these signals were further amplified at 16 h and continued unattenuated until 48 h (**Figures 6A and 6C**). These results indicated that Dex/PFP@LIPs-BMS-α could be efficiently targeted to the glomerulus and provides additional support for the application of LIFU therapy. We further analyzed the biodistribution of Dex/PFP@LIPs-BMS-α in PHN model rats (**Figure 6B**). There was no difference between heart and lung tissues with lower distribution of red DiI fluorescence. The spleen and liver had higher fluorescence signals after injection of Dex/PFP@LIPs compared to treatment with Dex/PFP@LIPs-BMS-α in PHN rats (**Figure 6D**). The results of these biodistribution experiments demonstrated that Dex/PFP@LIPs-BMS-α could be efficient and specific in targeting the kidney without being “trapped” by the liver and spleen *in vivo*.

We evaluated the targeting distribution in the glomeruli after Dex/PFP@LIPs-BMS-α combined with LIFU by TEM. As shown in **Figure S11A**, after intravenous injection of Dex/PFP@LIPs in PHN model rats, they were mainly distributed in the side of endotheliocyte and the side of Bowman's space (red circle), while they were widely distributed in podocytes after injection of Dex/PFP@LIPs-BMS-α (red circle). **Figure S11B** showed the TEM images of Dex/PFP@LIPs and Dex/PFP@LIPs-BMS-α for comparison. Notably, we observed vacuolated spherical matter (blue circle) in the PHN models, but not in the normal rat, which may be flocculent material, similar to intravascular plasma, and the dense round bodies 55. **Figure S11C** showed the foot process width after treatment. These results intuitively demonstrated that Dex/PFP@LIPs-BMS-α could cross the glomerular filtration barrier and reach the podocyte layer under the coordination of LIFU, and mainly targeted podocytes.

### Dex/PFP@LIPs-BMS-α with LIFU reduced ROS production *in vitro*

Reducing or blocking the production of reactive oxygen species in podocytes can alleviate podocyte injury 56,57. In our study, the productions of ROS (green) in podocytes were significantly observed after PA induction (**Figure S12A**), The levels of ROS in Dex and Dex with LIFU groups were lower than that of the control groups, as well as PFP@LIPs-BMS-α and PFP@LIPs-BMS-α with LIFU groups. The level of ROS in Dex/PFP@LIPs-BMS-α was significantly lower than that of the control group, especially after LIFU treatment (**Figure S12B**). These results suggested that the combination of Dex/PFP@LIPs-BMS-α and LIFU did reduce the level of ROS in podocytes.

### Treatment effectiveness and side-effects evaluation *in vivo*

**Figure 7:**
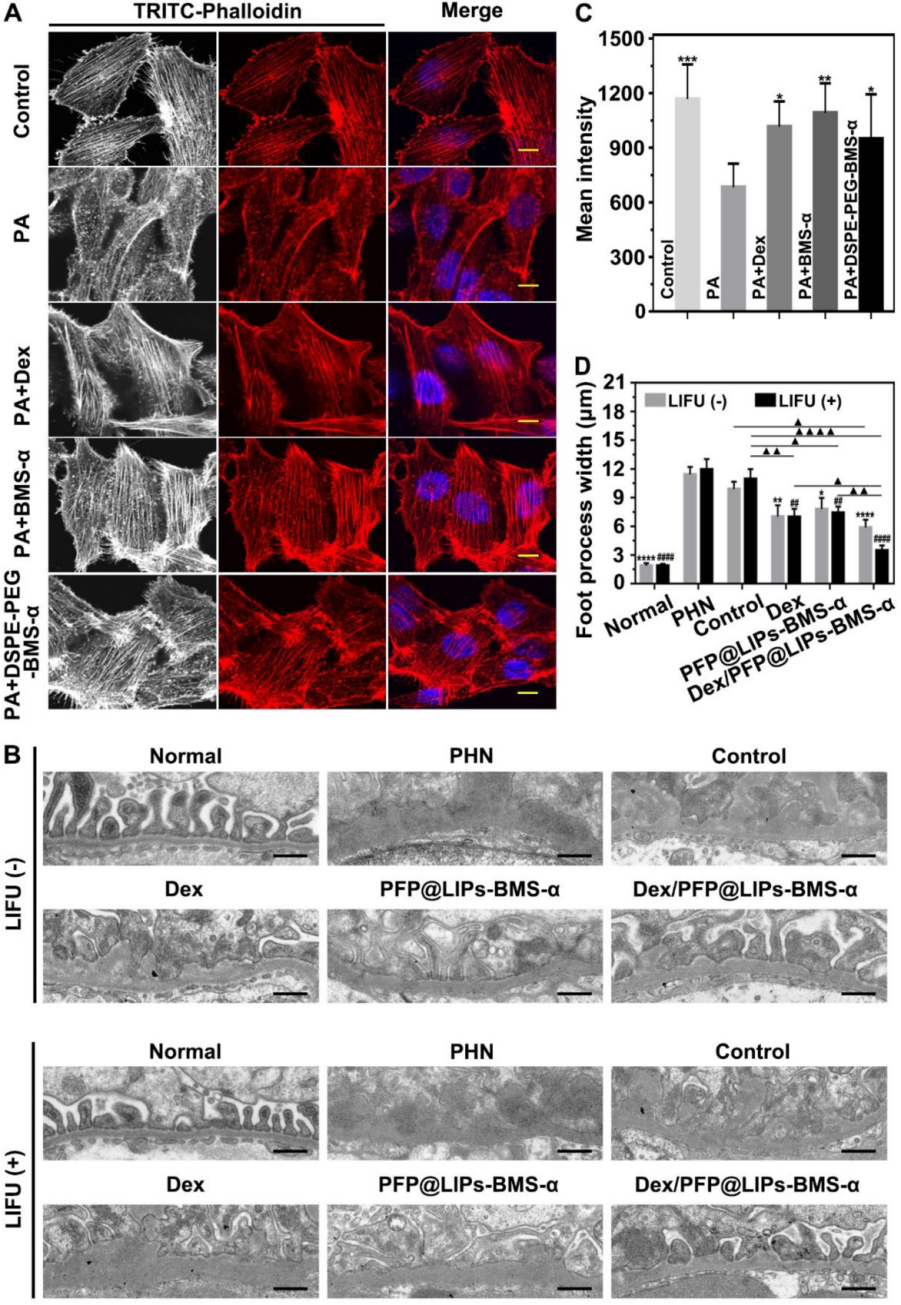
** Restoration of stress fibers after injury, and post-treatment evaluation according to the morphology of podocytes by TEM after various treatment *in vivo*.** (A) Stress fibers of human kidney podocytes were observed by CLSM after different treatment, including control, PA, PA + BMS-α and PA + DSPE-PEG-BMS-α. Red, black and white were the stress fibers, the nuclei were stained blue, scale bar = 10 µm. (B) The morphology of PHN rats' kidney podocytes were observed by TEM after 4 weeks of various treatment, and with LIFU or without LIFU. Saline was used as the control group, PHN model rats as the PHN groups, scale bar = 6 µm. (C). The mean fluorescence intensities were measured by a semi-quantitative method to evaluate restoration of stress fibers after injury, data are presented as mean ± SD, one-way ANOVA, *P < 0.05, ***P < 0.001, vs. PA group. (D) Quantitative analysis of the foot process width by Image J, data are presented as mean ± SD, one-way ANOVA, *^/#^*P <* 0.05, ****^/####^*P <* 0.0001, vs. control group.

The value of morphologically stabilizing the podocyte actin cytoskeleton to improve podocyte function in the treatment of nephritis has been demonstrated 10,11. Our DSPE-PEG-BMS-α was originally designed as the core “part” of the glucocorticoid nano-delivery system with active targeting function. It could also reduce the expression of MC-1R in podocytes and eliminate the damage induced by PA. In podocytes, the normal actin cytoskeleton presents a continuous and orderly arrangement. However, it collapses and fractures under the influence of renal inflammation. We used PA to induce podocyte damage to simulate nephritis and found that Dex, BMS-α and DSPE-PEG-BMS-α could reduce actin cytoskeleton injury induced by PA (**Figures 7A** and **7C**). The results indicated that BMS-α could stabilize the actin cytoskeleton, and supported as well by previous research 24,25, and lipid of BMS-α did not affect the role of BMS-α in protecting the actin cytoskeleton. In addition, Dex was demonstrated to stabilize actin cytoskeletons that directly protect podocytes, which have glucocorticoid receptors on their surfaces 14.

**Figure 8:**
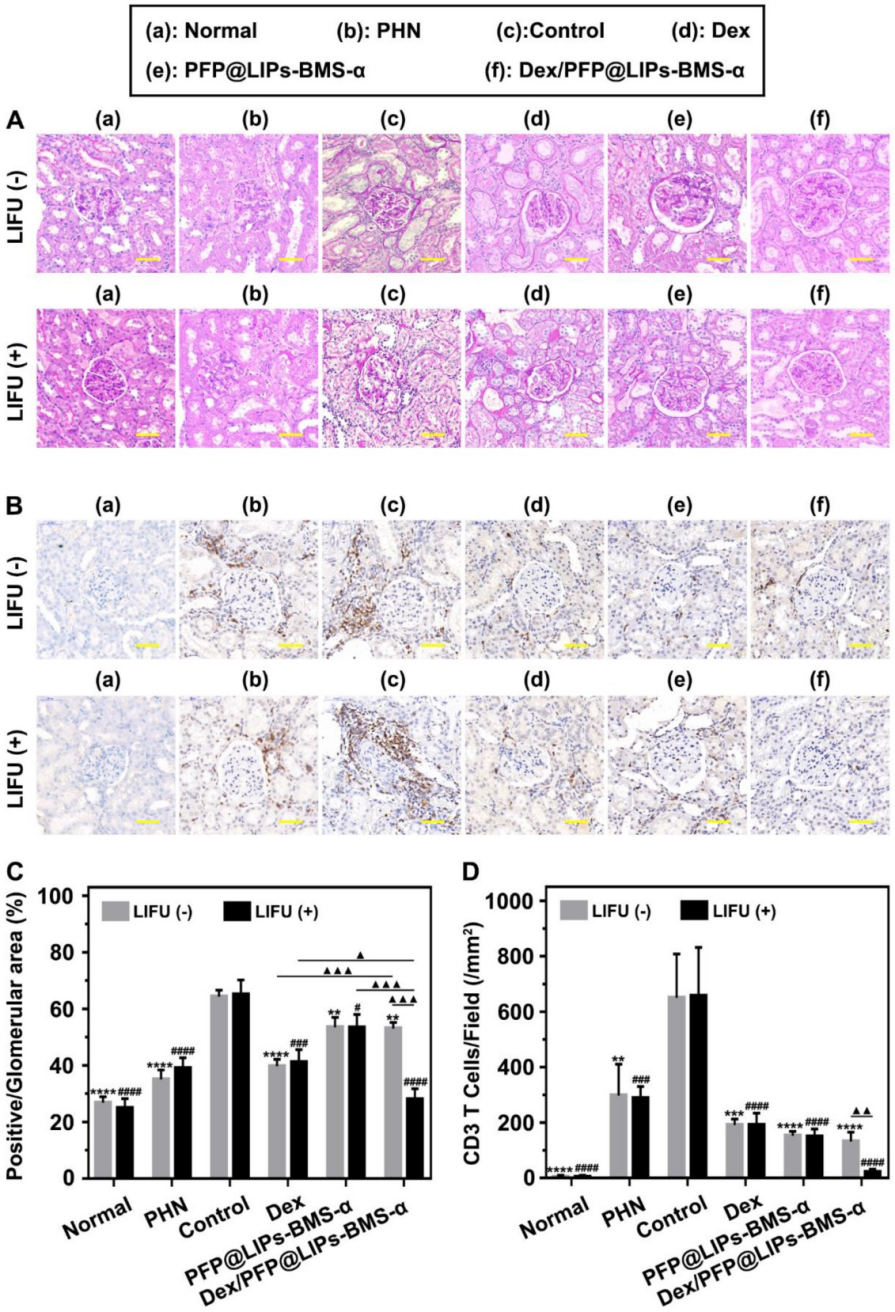
** Post-treatment evaluation according to PAS staining and IHC-P *in vivo*.** (A) PAS staining of PHN rats' kidneys after 4 weeks of various treatments, and with LIFU or without LIFU, scale bar = 50 µm. (B) Immunohistochemical staining of CD3 T-cells in renal tissue after 4 weeks of various treatments, scale bar = 50 µm. (C) Quantitative analysis of PAS staining results (Glomerular positive pixel area/Glomerular pixel area). (D) Immunohistochemical staining results of CD3 T-cells (Number of CD3 T-cells/Field). Saline was used as the control group, PHN model rats as the PHN groups. Data are presented as mean ± SD, one-way ANOVA, *^/#^*P <* 0.05, ****^/####^*P <* 0.0001, vs. control group. t-test, ^▲^*P <* 0.05, ^▲▲▲^*P <* 0.001.

TEM images have found podocyte foot process fusion accompanies basement membrane thickening of PHN rats (**Figure 7B**). Moreover, foot process width was almost normal after treatment with Dex/PFP@LIPs-BMS-α combined with LIFU for 4 weeks in PHN rats (**Figure 7D**). PAS staining showed glomerulosclerosis, basement membrane folds, balloon adhesions and tubular atrophy (**Figure 8A**). However, after 4 weeks of treatment, the morphology of the podocytes and glomeruli were reversed in the Dex group, Dex/PFP@LIPs-BMS-α group and Dex/PFP@LIPs-BMS-α with LIFU group. Pathologic changes in glomeruli and tubules were significantly improved after treatment with Dex/PFP@LIPs-BMS-α. Positive/total glomerular area analysis (**Figure 8C**) demonstrated that the structure had already been repaired to normal in Dex/PFP@LIPs-BMS-α with LIFU group. These results indicated that Dex/PFP@LIPs-BMS-α combined with LIFU could be used to improve podocyte morphology in immune-associated nephropathy.

Immunohistochemical staining analysis of CD3 T-cells/field (**Figure 8B**) showed that a large number of CD3 T-cells infiltrated the kidneys of both the PHN and control groups. After treatment with PFP@LIPs-BMS-α, Dex/PFP@LIPs-BMS-α, PFP@LIPs-BMS-α with LIFU and Dex/PFP@LIPs-BMS-α with LIFU for 4 weeks, CD3 T-cell infiltration was significantly reduced, especially in the Dex/PFP@LIPs-BMS-α with LIFU group (**Figure 8D**). These results suggested that Dex/PFP@LIPs-BMS-α with LIFU could effectively reduce the infiltration and expression of local inflammatory mediators in the kidney, and may play a role in *in-situ* immunosuppression of the kidney against nephritis.

Through the above actin cytoskeleton staining, TEM, PAS staining and immunohistochemistry (IHC) staining, we observed actin cytoskeleton rearrangement in podocytes after the PA induction, and that podocyte foot process fusion accompanying basement membrane thickening were observed by TEM in PHN rats. In addition, glomerulosclerosis and CD3 T-cell infiltration were apparent with PAS and IHC staining. These results suggested that the changes in podocyte morphology and function during nephritis were closely related to the occurrence of proteinuria. Podocyte injury during nephritis led to the actin cytoskeleton rearrangement, which further led to loss of the foot processes 58,59, also known as podocyte foot fusion 60. Moreover, foot fusion was closely related to the progression of glomerulosclerosis 61, which was specifically observed by PAS staining in the PHN rat. In fact, the above results indicated that dexamethasone nano-delivery system was effective in targeted therapy for repair of podocyte morphology and function.

**Figure 9:**
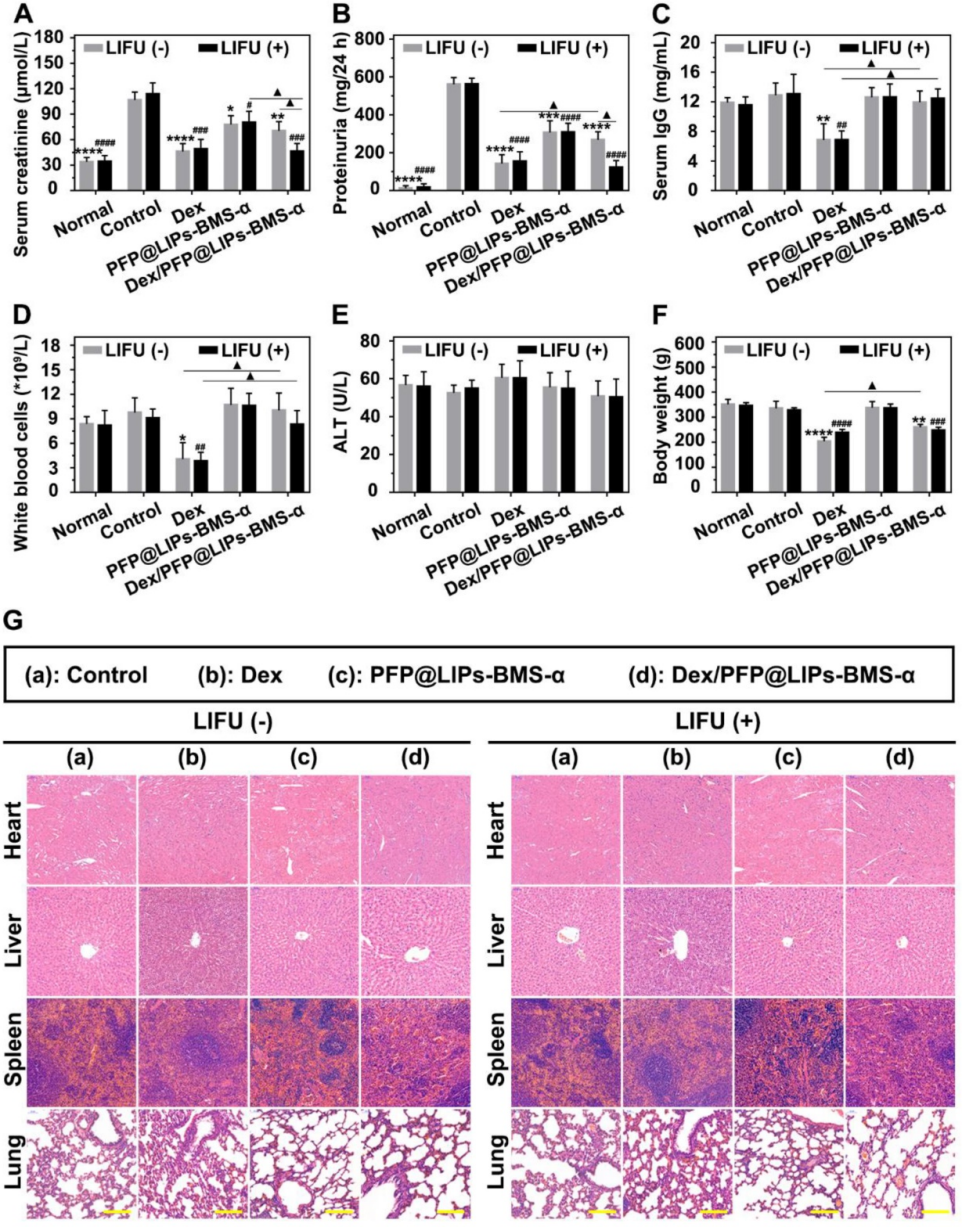
** Post-treatment evaluation according to treatment efficacy and safety assessment *in vivo*.** The levels of serum creatinine (A), proteinuria (B), serum IgG (C), white blood cells (D), alanine aminotransferase (ALT) (E) and body weight (F) after various treatment, and with LIFU or without LIFU. Saline was used as the control group. (G) The morphology of PHN rat's heart, liver, spleen and lung were observed by H&E staining after various treatment, and with LIFU or without LIFU, Saline was used as the control group, scale bar = 150 µm. Data are presented as mean ± SD, one-way ANOVA, *^/#^*P <* 0.05, ****^/####^*P <* 0.0001, vs. control group. t-test, ^▲^*P <* 0.05, ^▲▲^*P <* 0.01.

Serum creatinine and 24 h proteinuria levels were key indicators of the therapeutic effect on nephritis. As shown in**Figure S13A**-**B**, 24 h proteinuria levels were significantly increased after 14 days of PHN modeling, and persistently increased until the end in the control group. The PHN model appeared to provide stable high 24 h proteinuria levels. In the treatment group, 24 h proteinuria levels began to decrease 2-3 weeks after Dex + LIFU (-/+), PFP@LIPs-BMS-α + LIFU (-/+) and Dex/PFP@LIPs-BMS-α + LIFU (-/+). After 4 weeks of treatment, the serum creatinine (**Figure 9A**) and 24 h proteinuria levels (**Figure 9B**) of Dex + LIFU (-/+) groups, PFP@LIPs-BMS-α + LIFU (-/+) groups and Dex/PFP@LIPs-BMS-α + LIFU (-/+) groups were significantly lower than that of the control group.

At the beginning of Dex treatment, a decrease in serum IgG level was observed (**Figure S13C**-**D**). The serum IgG (**Figure 9C**) and WBC levels (**Figure 9D**) of the Dex group treated for 4 weeks were significantly lower than those of the control group, PFP@LIPs-BMS-α and Dex/PFP@LIPs-BMS-α with or without LIFU. These results evidence suggested that Dex had systemic immunosuppressive action. Targeted therapy by Dex/PFP@LIPs-BMS-α could effectively avoid such immunosuppression. Furthermore, no hepatotoxic injury (**Figure 9E**) was observed after treatment with Dex/PFP@LIPs-BMS-α with or without LIFU for 4 weeks.

Early body weight loss suggested a side effect in the Dex group, but gradually increased after treatment for a week in Dex/PFP@LIPs-BMS-α without LIFU group. No body weight loss was observed after treatment with Dex/PFP@LIPs-BMS-α combined with LIFU (**Figure S13E**-**F**). The body weight of the treatment group with Dex/PFP@LIPs-BMS-α combined with LIFU was greater than that of Dex group for 4 weeks (**Figure 9F**), indicating that Dex/PFP@LIPs-BMS-α combined with LIFU was relatively safe. In addition, no pathological changes were observed by H&E staining (**Figure 9G**) of the heart, liver, spleen and lung in each group. All of these results suggested that Dex/FPP@LIPs-BMS-α combined with LIFU was effective in treating immune-associated nephropathy and reduced the known side effects of systemic Dex.

In addition, the effect of dexamethasone nano-delivery system on glucose and lipid metabolism has been verified though different test indexes, including blood glucose, total cholesterol and triglyceride. As shown in **Figure S14**, during the observation period of 2 weeks, the blood glucose and blood lipid mentioned above showed no significant different in the PHN groups. Normal rats were used as the control groups. These results indicated that Dex/PFP@LIPs-BMS-α had no significant effect on glucose and lipid metabolism of nephropathy. On the other hand, a technology based on substrate materials, machine learning and laser desorption/ionization mass spectrometry has been developed to diagnose disease by extracting metabolic fingerprints 62,63. Unfortunately, renal metabolic profiling was difficult to achieve with our dexamethasone nano-delivery system. However, Dex/PFP@LIPs-BMS-α has showed excellent functions in drug delivery and image-based diagnosis with no effect on macro-metabolism mentioned above. These results were consistent with our original intention.

## Conclusion

In this study, we have built a new visualized podocyte-targeting and focused ultrasound responsive glucocorticoid nano-delivery system, Dex/PFP@LIPs-BMS-α combined with LIFU, and demonstrated its efficacy and safety as a new therapeutic strategy for immune-associate nephropathy. This new glucocorticoid nano-delivery system has a high affinity and specificity for the kidney, thereby avoiding the serious side effects of traditional systemic glucocorticoid treatment. Furthermore, the addition of LIFU to Dex/PFP@LIPs-BMS-α could provide visual diagnosis and treatment monitoring, including both ultrasound and photoacoustic, as well as actuate controllable drug release. These results present a promising and attractive approach for immune-associate nephropathy therapy.

## Supplementary Material

Supplementary figures.

## Abbreviations

Dex: dexamethasone
MC-1R: melanocortin-1 receptor
PFP: perfluoropentane
BMS-α: BMS-470539
UTMD: ultrasound-targeted microbubble destruction
LIFU: low intensity focused ultrasound
ADV: acoustic droplet vaporization
PEG: polyethylene glycol
DPPC: 1,2-dipalmitoyl-sn-glycero-3-phosphatidylcholine
DPPG: 1,2-dipalmitoyl-sn-glycero-3-phospho-(1-rac-glycerol)
Chol: cholesterol
DiI: 1,1'-Dioctadecyl-3,3,3',3'tetramethylindocarbocyanine perchlorate
DAPI: 2-(4-Amidinophenyl)-6-indolecarbamidinedihydrochloride
DiR: 1,1'-Dioctadecyl-3,3,3',3'-tetramethylindotricarbocyanine iodide
DSP: dexamethasone sodium phosphate
PA: puromycin aminonucleoside
CCK-8: cell counting kit-8
HPLC: high-performance liquid chromatography
Anti-Fx1A: sheep anti-rat Fx1A serum
DMF: N'N-dimethyl formamide
HOBt: anhydrous 1-hydroxybenzotriazole
DIC: N'N-diisopropylcarbodiimide
PHN: passive heymann nephritis
ROS: reactive oxygen species
TEM: transmission electron microscope
FBS: fetal bovine serum
CLSM: confocal laser scanning microscope
WBC: white blood cells
ALT: glutamic-pyruvic transaminase
H&E: hematoxylin and eosin
PAS: periodic Acid-Schiff
IHC: immunohistochemistry
PAI: photoacoustic imaging
