# Supplementary material for: Visualized podocyte-targeting and focused ultrasound responsive glucocorticoid nano-delivery system against immune-associated nephropathy without glucocorticoid side effect
Source: Theranostics. 2021 Jan 1;11(6):2670–90. doi: 10.7150/thno.53083 (PMC7806481; doi:10.7150/thno.53083)
Supplement: Supplementary file 1 — Supplementary figures. [file thnov11p2670s1.pdf]

Supporting Information

Visualized podocyte-targeting and focused ultrasound responsive glucocorticoid nano-delivery system against immune-associated nephropathy without glucocorticoid side effect

Kui Fan[1,2,3#], Li Zeng[1,2,4#*], Jing Guo[5], Shuqin Xie[1], Yuan Yu[1], Jianwei Chen[1], Jin Cao[2], Qinyanqiu Xiang[2], Siliang Zhang[1], Yuanli Luo[2], Qingyue Deng[1], Qin Zhou[1],Yan Zhao[2,6], Lan Hao[2], Zhigang Wang[2*], Ling Zhong[1*]

1. Department of Nephrology, The Second Affiliated Hospital of Chongqing Medical University, Chongqing 400010, China

2. Institute of Ultrasound Imaging, The Second Affiliated Hospital of Chongqing Medical University, Chongqing 400010, China

3. Department of Nephrology, Santai County People's Hospital (Affiliated Hospital of North Sichuan Medical College in Santai County), Mianyang 621100, China

4. State Key Laboratory of Ultrasound in Medicine and Engineering, Chongqing Medical University, Chongqing 400016, China

5. Radiation Oncology Center, Chongqing University Cancer Hospital, Chongqing University, Chongqing 400030, China

6. Department of Respiratory Medicine, The Second Affiliated Hospital of Chongqing Medical University, Chongqing 400010, China

#The authors contributed equally to this work.

* These authors are co-corresponding authors.

Corresponding authors: E-mail: zhongling@hospital.cqmu.edu.cn (Ling Zhong), 303507@hospital.cqmu.edu.cn (Zhigang Wang), zengli@cqmu.edu.cn (Li Zeng)

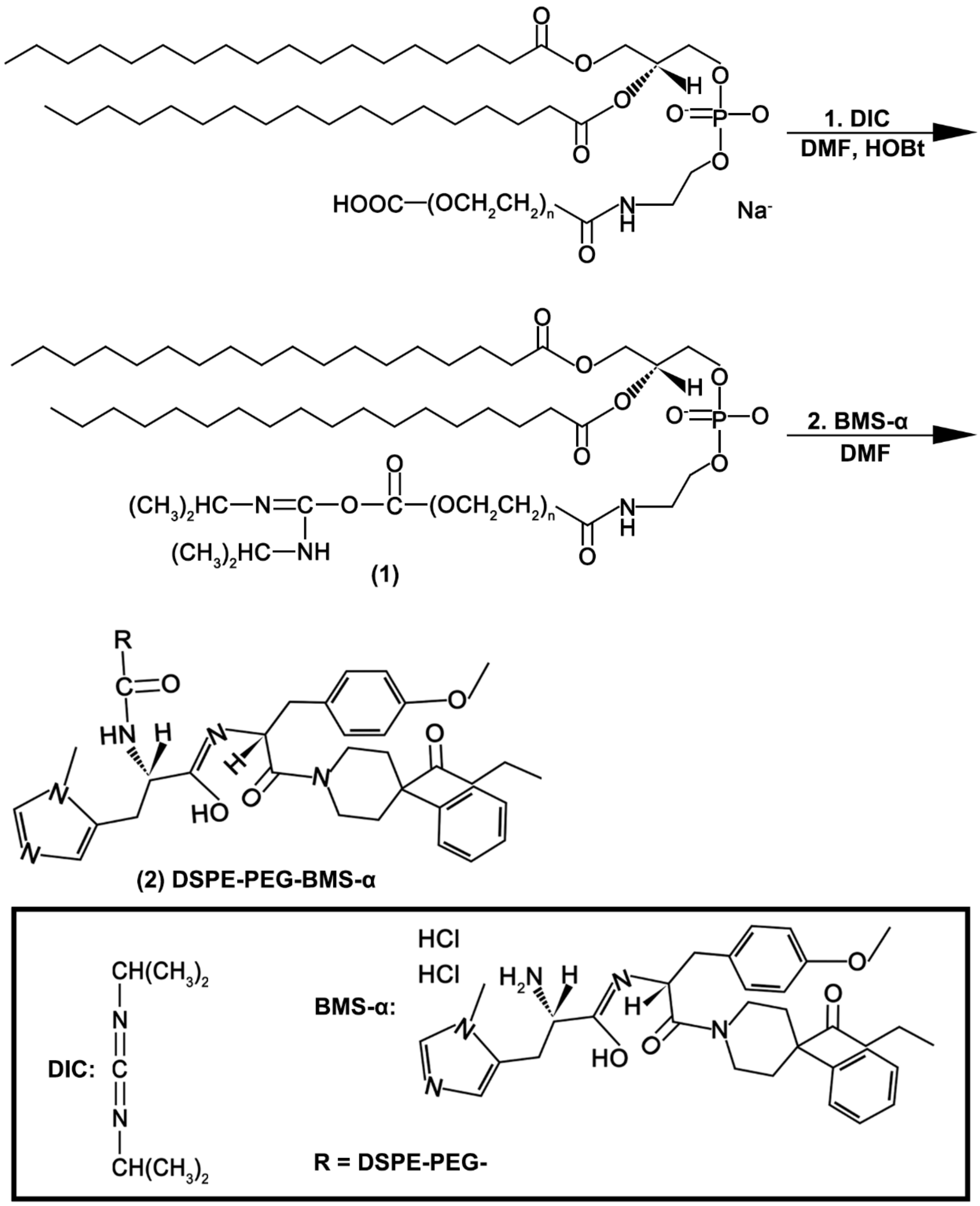

Figure S1. Synthetic process of the DSPE-PEG-BMS-α. Reagents and conditions: 1. 80 mg DSPE-PEG-COOH, 20 mL DMF, 3.8 mg HOBt, 3.5 mg DIC, rt, 30 min; 2.15 mg BMS-α, 2 mL DMF, rt, 2 h.

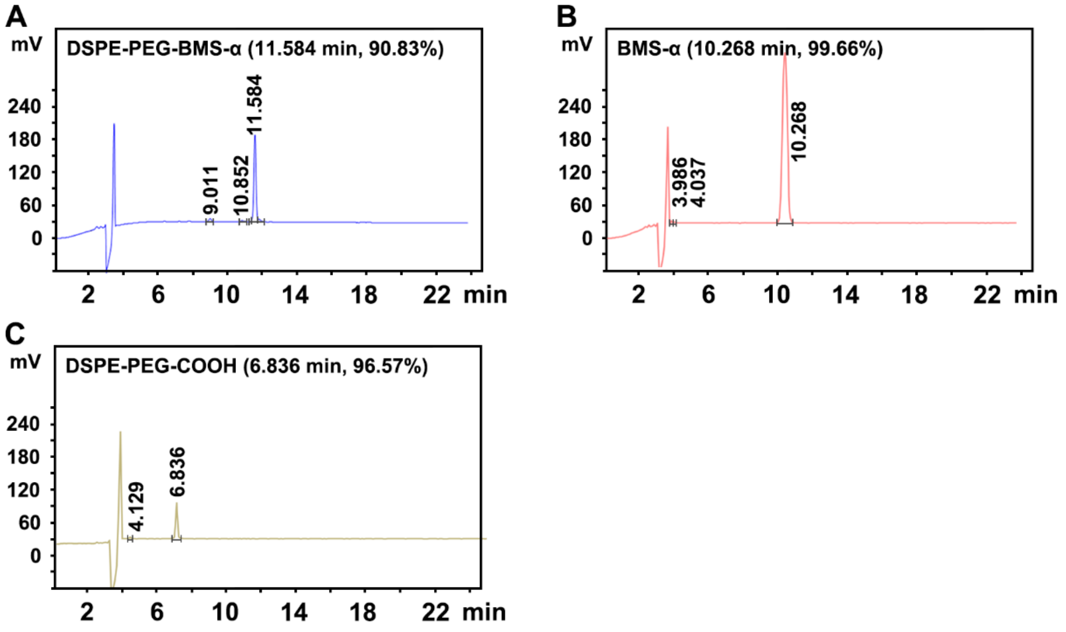

Figure S2. HPLC of BMS-α, DSPE-PEG-COOH and DSPE-PEG-BMS-α.

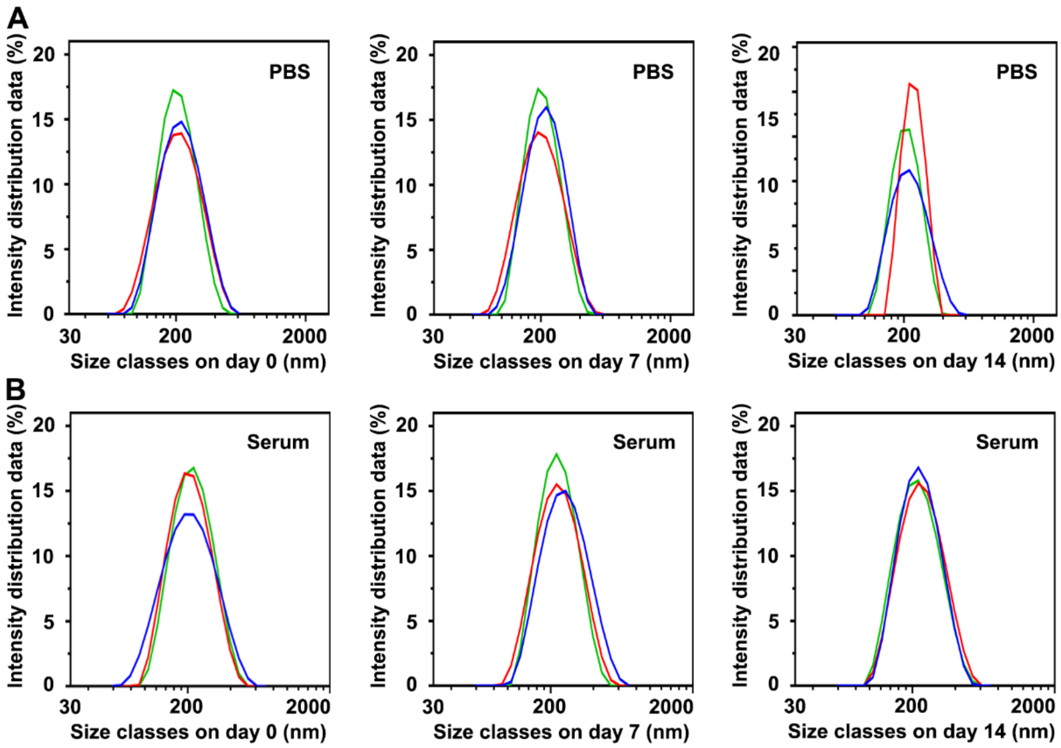

Figure S3. The stability of Dex/PFP@LIPs-BMS-α. The size distribution of Dex/PFP@LIPs-BMS-α at 0 d, 7 d and 14 d in PBS (4 ℃) (A) and serum (room temperature) (B).

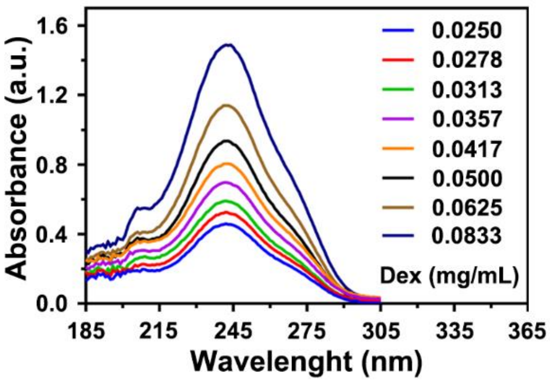

Figure S4. The UV-vis absorption spectra of free Dex. The UV-vis absorption spectrum of free Dex at 185-365 nm under different concentrations. The characteristic absorption peak appeared at 242 nm.

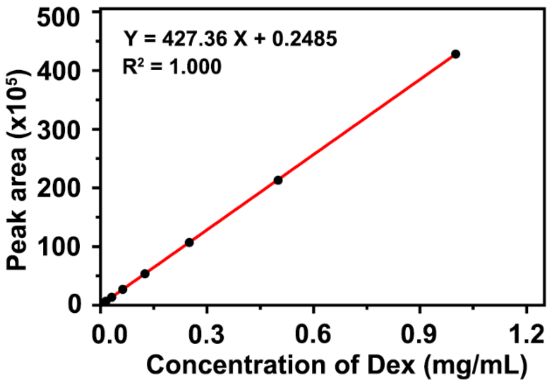

Figure S5. The standard curve of Dex constructed form HPLC measurements.

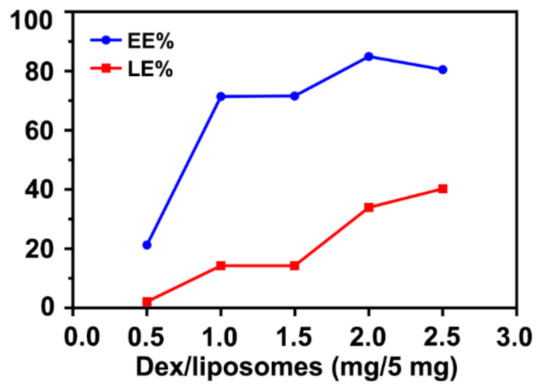

Figure S6. HPLC determined the EE% and LE % of Dex. The EE% and LE% of Dex in Dex/PFP@LIPs-BMS-α were determined by HPLC.

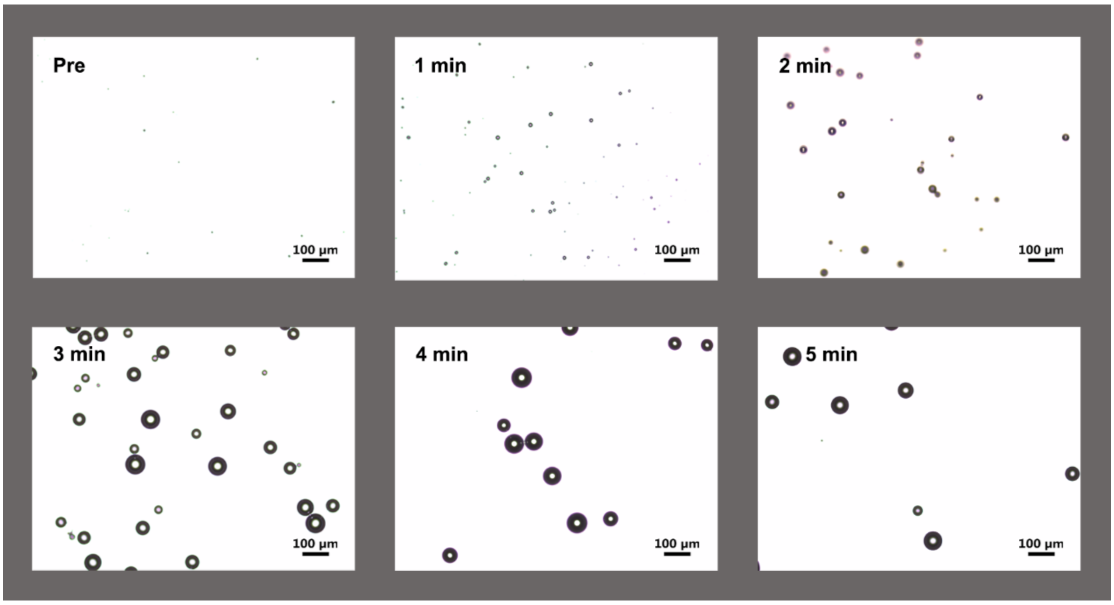

Figure S7. ***In vitro*** **the time-dependent liquid-gas phase transition process of Dex/PFP@LIPs-BMS-α after LIFU irradiation (2.4 W/cm^2, 1 MHz, duty cycle of 50%).**

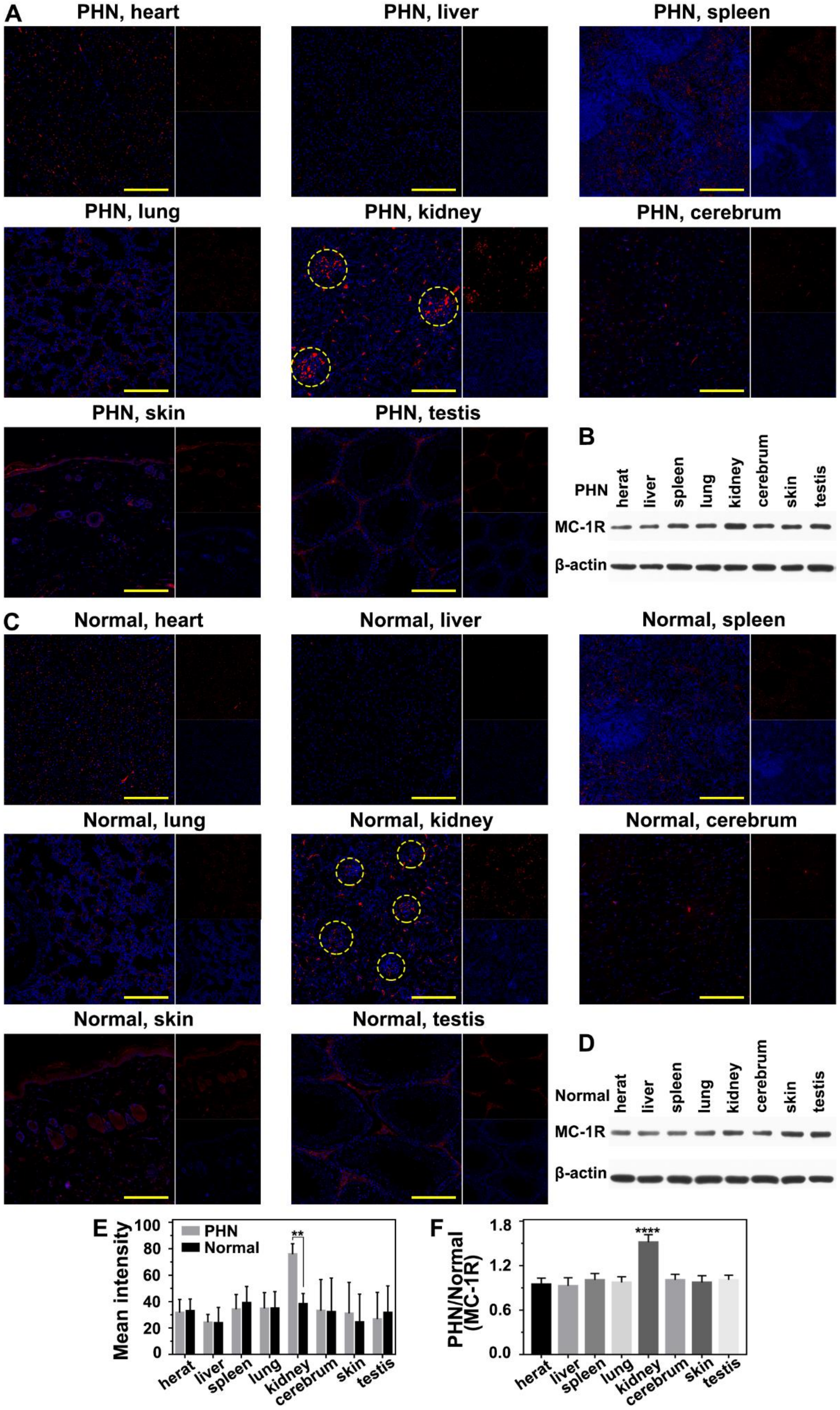

A
PHN, heart
PHN, liver
PHN, spleen
PHN, lung
PHN, kidney
PHN, cerebrum
PHN, skin
PHN, testis
B
PHN
herat
liver
spleen
lung
kidney
cerebrum
skin
testis
MC-1R
β-actin
C
Normal, heart
Normal, liver
Normal, spleen
Normal, lung
Normal, kidney
Normal, cerebrum
Normal, skin
Normal, testis
D
Normal
herat
liver
spleen
lung
kidney
cerebrum
skin
testis
MC-1R
β-actin
E
Mean intensity
PHN
Normal
**
0
20
40
60
80
100
herat
liver
spleen
lung
kidney
cerebrum
skin
testis
F
PHN/Normal (MC-1R)
****
0.0
0.6
1.2
1.8
herat
liver
spleen
lung
kidney
cerebrum
skin
testis

Figure S8. The expression levels of MC-1R in PHN and normal rats' organs. The expression levels of MC-1R in PHN model rat's organs were detected by immunofluorescence (A) and WB (B). The expression levels of MC-1R in normal rat's organs were detected by immunofluorescence (C) and WB (D). (E) The results of mean fluorescence intensity. (F) The WB analysis of MC-1R in PHN model and normal rats' organs. Scale bar = 200 μm, the yellow circles show the glomeruli. Data are presented as mean ± SD, one-way ANOVA, $**P < 0.01$, $****P < 0.0001$.

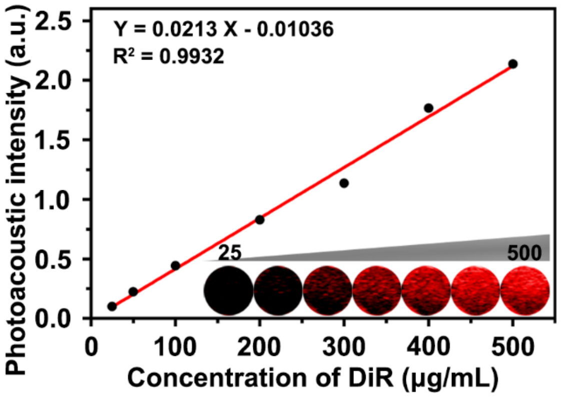

Figure S9. The DiR dose-dependent photoacoustic intensity curve, and the PAI of DiR-labeled Dex/PFP@LIPs-BMS-α *In vitro*. The photoacoustic intensity and PAI of Dex/PFP@LIPs-BMS-α under different DiR concentrations.

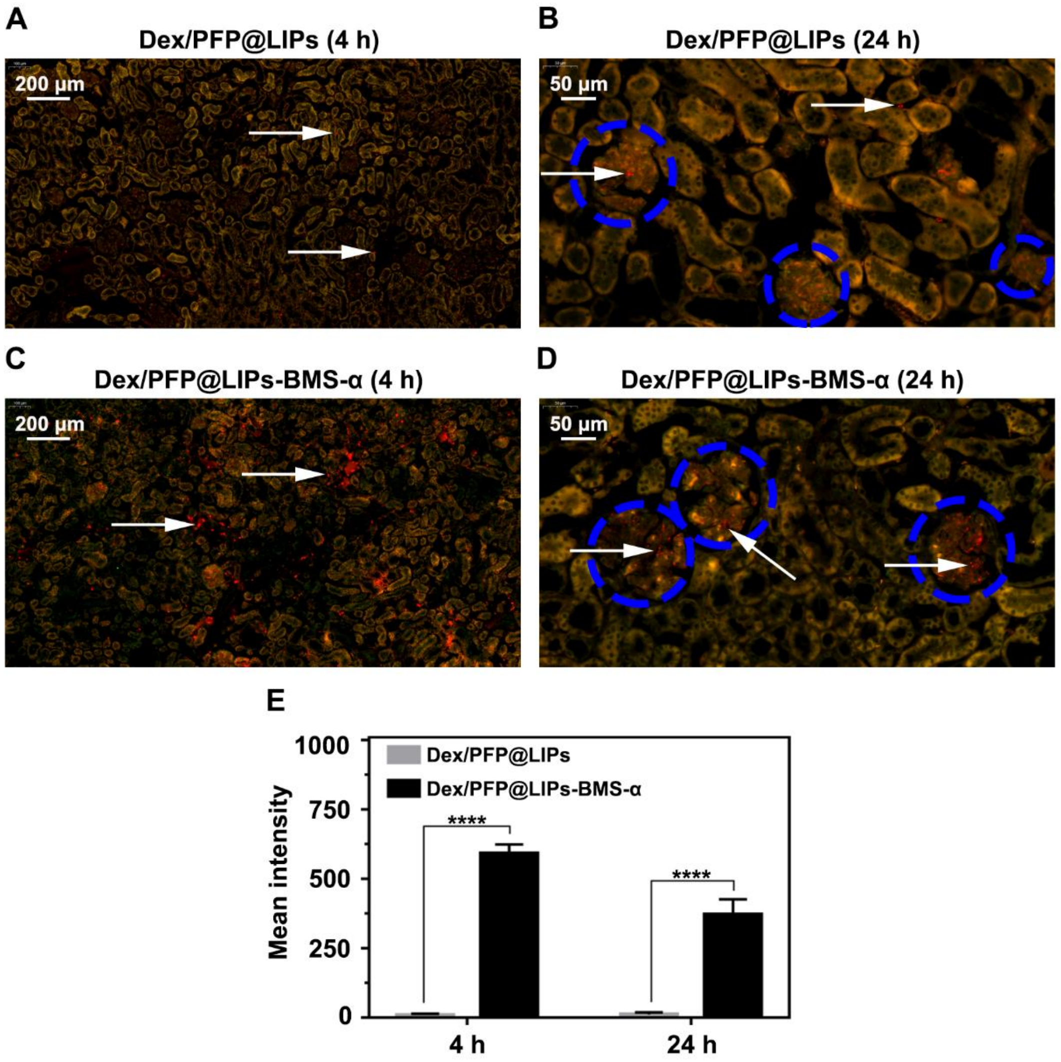

Figure S10. Kidney targeting efficiency of Dex/PFP@LIPs-BMS-α by cells localization *in vivo*. (A) *In vivo* efficiency of Dex/PFP@LIPs-BMS-α by cells localization at various intervals (4 and 24 h). yellow was self-luminous, and the blue circles indicate the glomeruli. The red dots (white arrows) were the Dex/PFP@LIPs or Dex/PFP@LIPs-BMS-α (labeled by DiI). (B) The mean fluorescence intensities were measured by a semi-quantitative method. Data are presented as mean ± SD, t-test, $****P < 0.0001$.

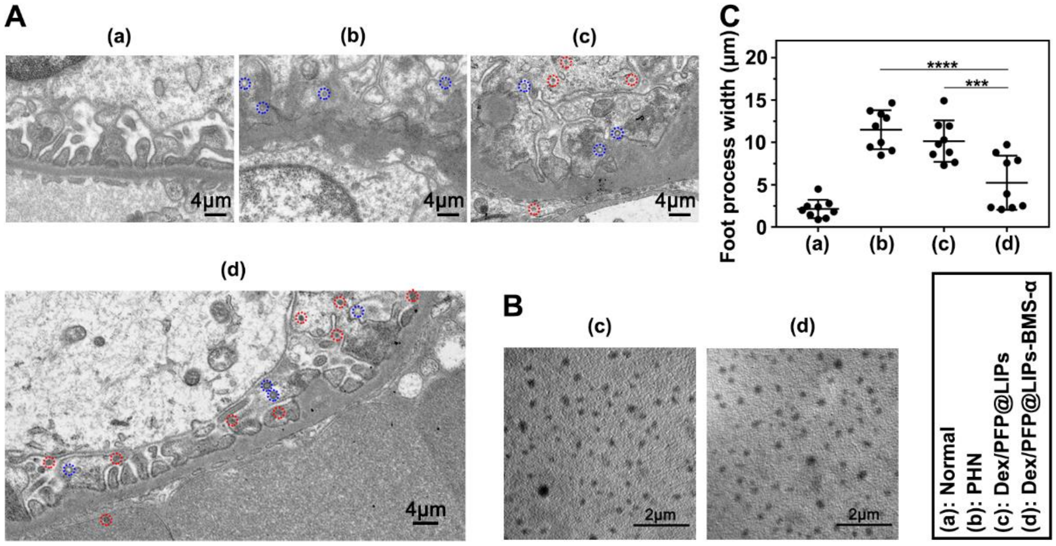

Figure S11. Renal targeting efficiency of Dex/PFP@LIPs-BMS-α. (A) The targeted distribution was observed by TEM in the glomeruli after Dex/PFP@LIPs (red circle) or Dex/PFP@LIPs-BMS-α (red circle), and which treated with LIFU. Vacuolated spherical matter (blue circle) may be a flocculent material, similar to the intravascular plasma and the dense round bodies. (B) The TEM images of Dex/PFP@LIPs and Dex/PFP@LIPs-BMS-α for comparison. (C) Statistical analysis of the foot process width. Data are presented as mean ± SD. one-way ANOVA, ***P < 0.001, ****P < 0.0001.

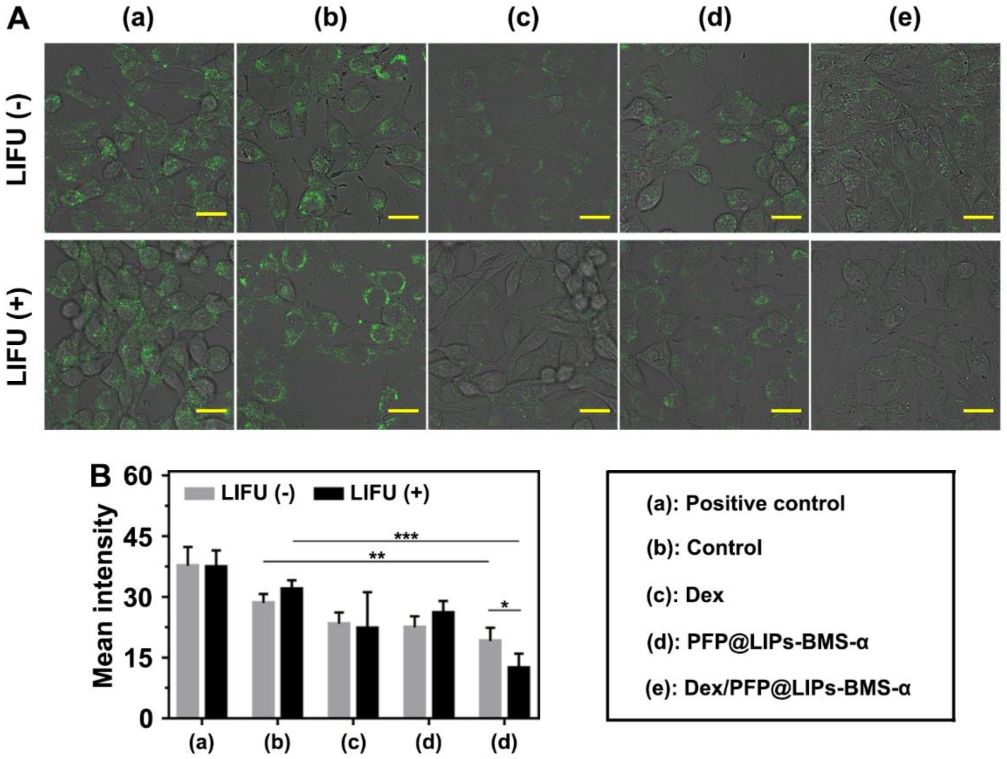

Figure S12. Dex/PFP@LIPs-BMS-α with LIFU reduced ROS production in vitro. (A) The ROS of podocytes were determined by DCFDA (green) after co-incubated with PBS (control), Dex, PFP@LIPs-BMS-α and Dex/PFP@LIPs-BMS-α, with or without LIFU, scale bar = 25 μm. (B) The mean fluorescence intensity of ROS was measured by a semi-quantitative at the corresponding point in time. Data are presented as mean ± SD. one-way ANOVA, **P* < 0.05, ****P* < 0.001.

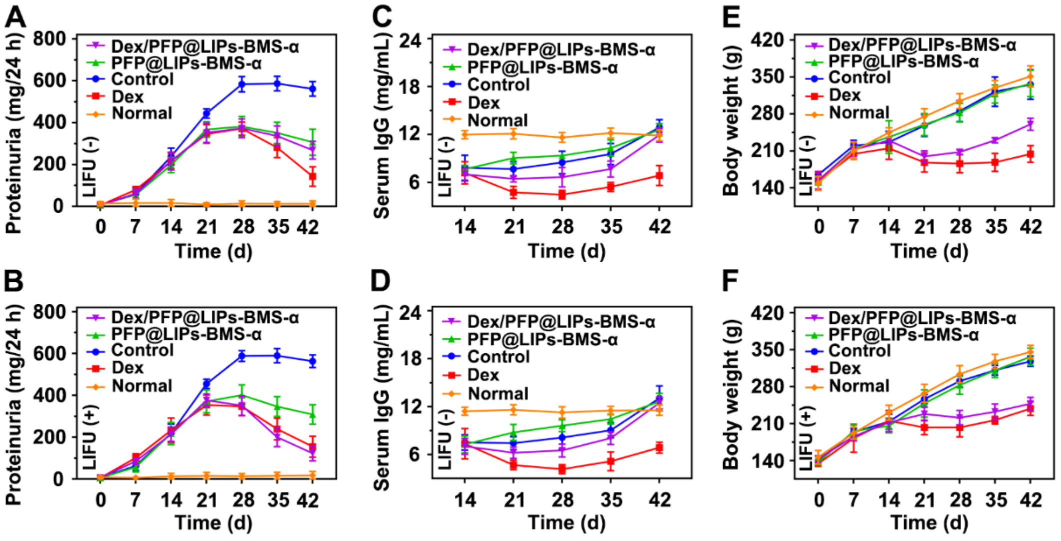

Figure S13. Whole-process tracking evaluation of treatment. The proteinuria levels were measured after various treatment without LIFU (A) or with LIFU (B). Supplementary instruction, the first 14 days were the model building period, n = 4-5. The serum IgG levels were determined from 14th to 42th after various treatment without LIFU (C) or with LIFU (D). The body weight indicators were recorded from 0th to 42th after various treatment without LIFU (E) or with LIFU (F). Data are presented as mean ± SD, one-way ANOVA.

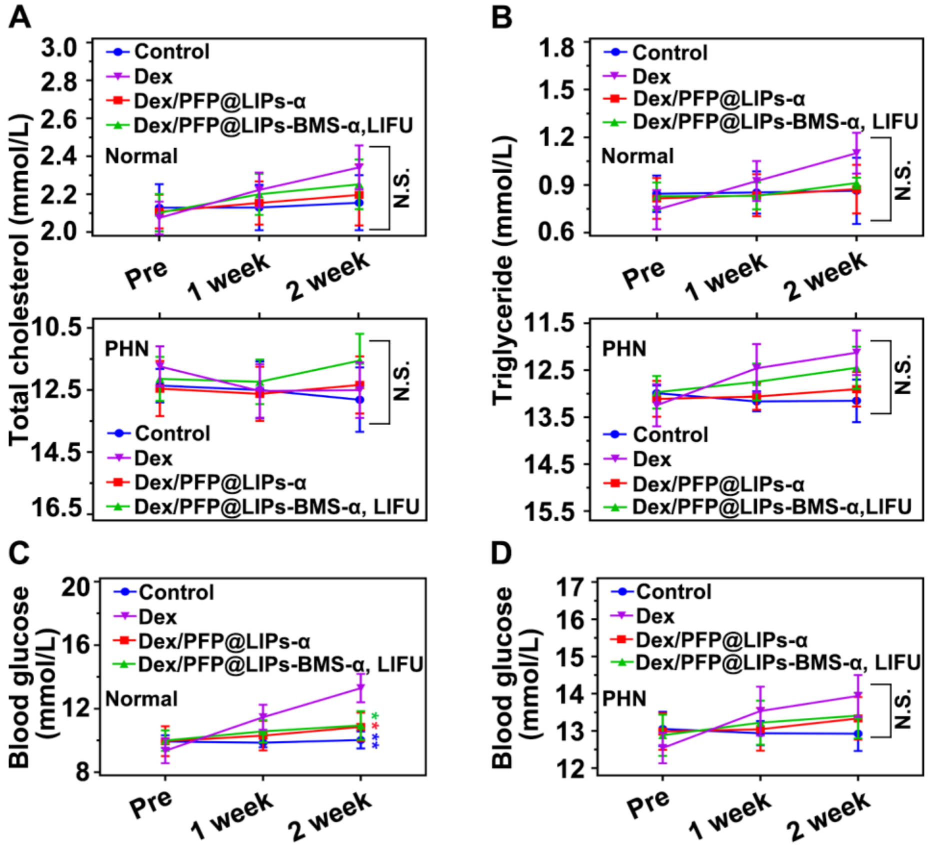

Figure S14. Lipid and glucose metabolism. (A) Total cholesterol levels in normal and PHN rats. (B) Triglyceride levels in normal and PHN rat. (C) Blood glucose levels in normal and PHN rat. Data are presented as mean ± SD, one-way ANOVA, **P* < 0.05, ***P* < 0.01, ****P* < 0.001 vs. Dex group.
